# Effect of polymorphisms in drug metabolism and transportation on plasma concentration of atorvastatin and its metabolites in patients with chronic kidney disease

**DOI:** 10.3389/fphar.2023.1102810

**Published:** 2023-02-27

**Authors:** Zebin Jiang, Zemin Wu, Ruixue Liu, Qin Du, Xian Fu, Min Li, Yongjun Kuang, Shen Lin, Jiaxuan Wu, Weiji Xie, Ganggang Shi, Yanqiang Peng, Fuchun Zheng

**Affiliations:** ^1^ Clinical Pharmacology Laboratory, First Affiliated Hospital of Shantou University Medical College, Shantou, China; ^2^ Department of Pharmacology, Shantou University Medical College, Shantou, China; ^3^ Department of Anesthesiology, Second Affiliated Hospital of Shantou University Medical College, Shantou, China; ^4^ Department of Nephrology, Second Affiliated Hospital of Shantou University Medical College, Shantou, China; ^5^ Department of Nephrology, First Affiliated Hospital of Shantou University Medical College, Shantou, China

**Keywords:** atorvastatin, genetic polymorphism, drug-metabolizing enzymes, transporters, plasma concentration, ABCC4

## Abstract

Dyslipidemia due to renal insufficiency is a common complication in patients with chronic kidney diseases (CKD), and a major risk factor for the development of cardiovascular events. Atorvastatin (AT) is mainly used in the treatment of dyslipidemia in patients with CKD. However, response to the atorvastatin varies inter-individually in clinical applications. We examined the association between polymorphisms in genes involved in drug metabolism and transport, and plasma concentrations of atorvastatin and its metabolites (2-hydroxy atorvastatin (2-AT), 2-hydroxy atorvastatin lactone (2-ATL), 4-hydroxy atorvastatin (4-AT), 4-hydroxy atorvastatin lactone (4-ATL), atorvastatin lactone (ATL)) in kidney diseases patients. Genotypes were determined using TaqMan real time PCR in 212 CKD patients, treated with 20 mg of atorvastatin daily for 6 weeks. The steady state plasma concentrations of atorvastatin and its metabolites were quantified using ultraperformance liquid chromatography in combination with triple quadrupole mass spectrometry (UPLC−MS/MS). Univariate and multivariate analyses showed the variant in ABCC4 (rs3742106) was associated with decreased concentrations of AT and its metabolites (2-AT+2-ATL: β = -0.162, *p* = 0.028 in the dominant model; AT+2-AT+4-AT: β = -0.212, *p* = 0.028 in the genotype model), while patients carrying the variant allele ABCC4-rs868853 (β = 0.177, *p* = 0.011) or NR1I2-rs6785049 (β = 0.123, *p* = 0.044) had higher concentrations of 2-AT+2-ATL in plasma compared with homozygous wildtype carriers. Luciferase activity was enhanced in HepG2 cells harboring a construct expressing the rs3742106-T allele or the rs868853-G allele (*p* < 0.05 for each) compared with a construct expressing the rs3742106G or the rs868853-A allele. These findings suggest that two functional polymorphisms in the ABCC4 gene may affect transcriptional activity, thereby directly or indirectly affecting release of AT and its metabolites from hepatocytes into the circulation.

## 1 Introduction

China has one of the largest populations of patients with chronic kidney diseases (CKD) in Asia (up to 159.8 million in 2020) (Liyanage et al., 2022). Dyslipidemia due to renal insufficiency is a common complication in patients with CKD, leads to further development of kidney damage and deterioration of kidney function (Jungers et al., 1997). Both CDK and dyslipidemia are considered to be major risk factors for cardiovascular events (Sarnak et al., 2003; Kopin and Lowenstein, 2017). Therefore, for CKD patients, treatment of dyslipidemia is particularly important.

Atorvastatin (AT) is currently the first-line drug for lipid lowering and prevention of cardiovascular disease (Arca and Gaspardone, 2007). As an HMG-CoA reductase inhibitor, atorvastatin reduces cholesterol synthesis and increases the number of LDL receptors on the surface of hepatocytes, thereby reducing plasma LDL cholesterol levels, and has a stronger lipid-lowering effect in women or in patients with non-familial hyperlipidemia (Adams et al., 2015). In addition to its lipid-lowering effects, atorvastatin also protects the cardiovascular system and reduces damage to the kidney through its multiple anti-inflammatory, antioxidative, endothelial protective and anti-cell proliferative effects (Aviram et al., 1998; Mason, 2006). CKD patients who are not on dialysis or renal transplantation were are now recommended to adopt lipid-regulating therapy with statins, according to the Kidney Disease Improving Global Outcomes (KDIGO) Clinical Practice Guideline (Tonelli and Wanner, 2014).

However, there are significant individual differences in clinical responses (efficacy or toxicity) to atorvastatin among different patients. Previous studies have reported that nearly one-third of patients fail to achieve lipid-lowering goals despite dose adjustments to statins based on patient response to treatment (Mangravite et al., 2006). Observational studies have found that 10%–15% of statin users experience varying degrees of statin-related muscle side effects, including mild muscle pain, muscle cramps, muscle weakness and even the rare and serious symptom of rhabdomyolysis (Abd and Jacobson, 2011). This individual variation in pharmacodynamics is related to the level of drug in the plasma (Link et al., 2008). However, the plasma AT levels are not only influenced by clinical factors such as gender, age, BMI, co-morbidities and co-administration (Turner et al., 2020a; Hirota et al., 2020), but also are strongly associated with polymorphisms in genes related to drug absorption, distribution, metabolism and excretion (ADME) (DeGorter et al., 2013; Cruz-Correa et al., 2017; Turner et al., 2020b) which have been reported to have high inter-individual variability (45-fold) (DeGorter et al., 2013). It is also noteworthy that genetic variability may contribute to >90% of the variance in plasma AT concentrations, and mainly occurs in genes for drug metabolizing enzymes and transporters (DeGorter et al., 2013; Cruz-Correa et al., 2017; Turner et al., 2020b).

**FIGURE 1:**
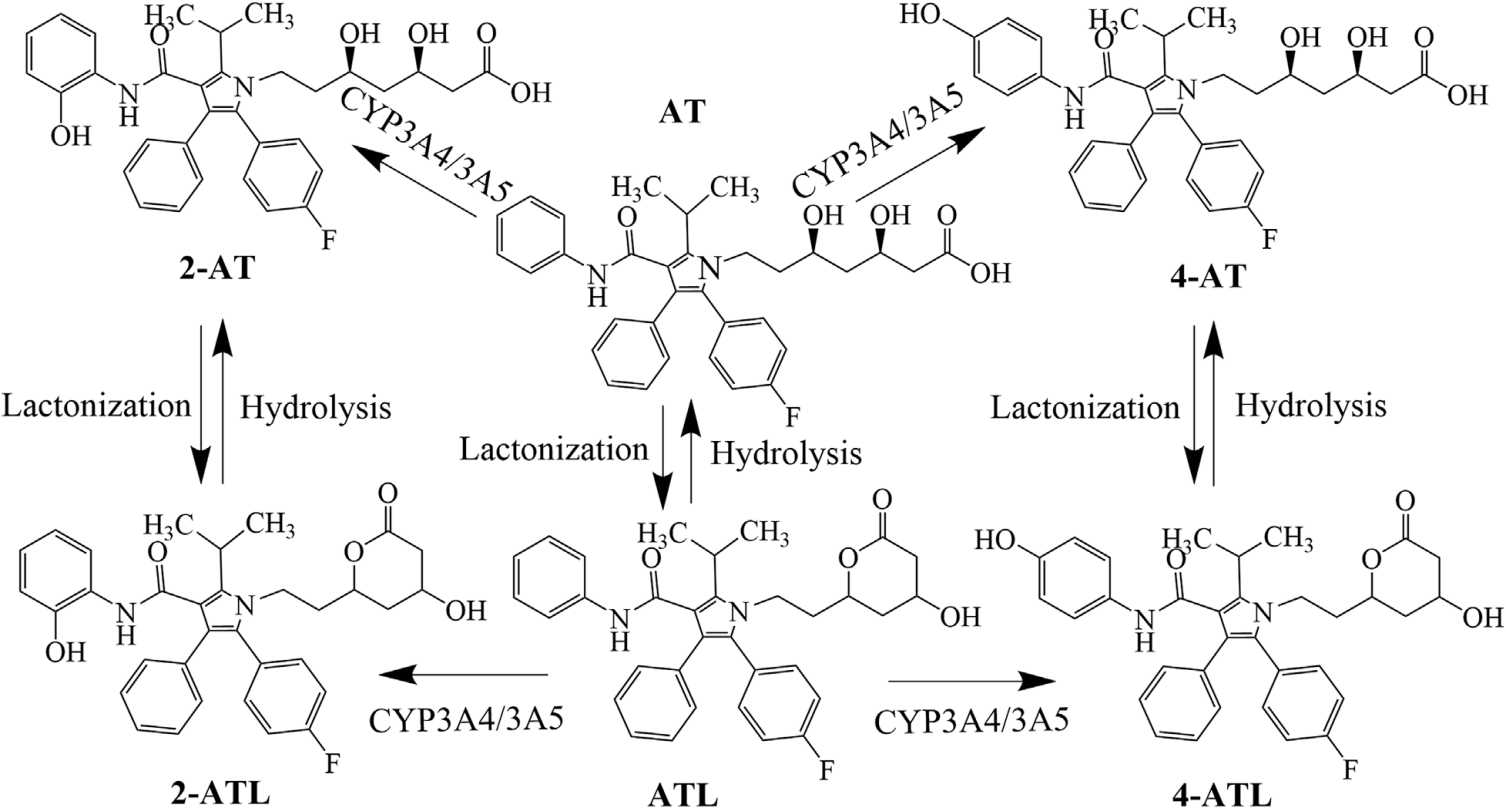
Conversion processes of atorvastatin and its metabolites.

AT is administered orally as a calcium salt that is absorbed into the blood *via* the small intestine and taken up by the OATP transporter into hepatocytes, where it is metabolized by *CYP3A4* and *CYP3A5* to the partially bioactive hydroxylated derivatives (2-hydroxy atorvastatin (2-AT) and 4-AT) (Lennernäs, 2003). AT and its active metabolites undergo lactonization *via* an unstable acyl glucuronide intermediate to produce AT lactone (ATL), 2-ATL and 4-ATL (Prueksaritanont et al., 2002). These lactone metabolites can be hydrolyzed to the corresponding hydroxy acids by plasma paraoxonases or by pH changes (Riedmaier et al., 2011) (Figure 1). AT and its metabolites are mainly eliminated by bile, with only about 1% excreted by the kidneys (Lennernäs, 2003). Its hydroxylated metabolite was found to inhibit HMG-CoA reductase as much as AT *in vitro*, about 70% of the total plasma HMG-CoA reductase inhibitory activity is accounted for by active metabolites (Lea and McTavish, 1997; Prake-Davis, 2004). In addition, the active metabolites of AT also protect the cardiovascular system with pleiotropic effects such as anti-oxidation and improvement of endothelial function.^[20, 21]^ Therefore, the plasma concentration of AT metabolites should also be of concern.

*CYP3A4* and *CYP3A5* enzymes are the main metabolizing enzymes for AT, playing an important role in disposition of AT. It has been reported that rs2242480, located on intron 10 of *CYP3A4*, is associated with the lipid-lowering efficacy of atorvastatin (Gao et al., 2008; Peng et al., 2018), and the presence of rs4646437 in *CYP3A4* intron 7 affects *CYP3A4* protein expression and enzymatic activity in a gender-dependent manner (Schirmer et al., 2007), but there has been no study on the effects of these polymorphisms on plasma AT concentration. CYP3A5*3 (rs776746C) in intron 3 of *CYP3A5* leads to aberrant splicing of mRNA and truncation of protein, resulting in deletion of CYP3A5 protein in individuals homozygous for this allele (Kuehl et al., 2001), and has been shown to be associated with increased systemic atorvastatin in a prior bioequivalence study in healthy volunteers (Zubiaur et al., 2021).

The OATP1B1 transporter, encoded by *SLC O 1B1* (König et al., 2000), mediates the absorption of drugs into hepatocytes, and is the rate-limiting step in the hepatic clearance of atorvastatin (Maeda et al., 2011). Previous studies have revealed that single coding single-nucleotide polymorphisms (SNPs, rs4149056 and rs2306283) of *SLC O 1B1* are associated with the plasma AT concentration (DeGorter et al., 2013). Moreover, *SLC O 1B1* rs4149056 contributes to statin-induced myopathy, and the Clinical Pharmacogenetics Implementation Consortium (CPIC) has published guidelines for the use of atorvastatin in carriers of this SNP (Cooper-DeHoff et al., 2022), but it is not specifically indicated for CKD complications.

Atorvastatin is a substrate for the efflux transporters P-glycoprotein, encoded by ABCB1, and BCRP, encoded by ABCG2, which may limit intestinal absorption and biliary clearance of AT. The common SNPs of *ABCB1* (rs1045642, rs1128503 and rs2032582) have been reported to be associated with the lipid-lowering efficacy of atorvastatin (Thompson et al., 2005; Hoenig et al., 2011; Prado et al., 2018), but their association with plasma AT concentration is unknown. The *ABCG2* (421C>A) variant (rs2231142) contains a replacement of glutamine with lysine at position 141 in the intracellular region of the protein, and demonstrates lower protein expression and transport capacity in cells transfected with the variant than with the wild type (Imai et al., 2002; Kondo et al., 2004). Although many studies have shown a correlation between rs2231142 and plasma concentrations of atorvastatin, the phenotypic results of the effects differ in different populations (Birmingham et al., 2015; Lee et al., 2019).

In addition to the above transporters, MRP3, MRP4 and MRP5, which are localized in the basolateral membrane of hepatocytes, have also been found to be involved in the transport of atorvastatin *in vitro* (Knauer et al., 2010; Deng et al., 2021), and their polymorphisms may play an important role in the plasma concentration of AT and its metabolites. Previous studies have indicated that rs4793665 located in the *ABCC3* promoter region, affects the plasma concentration of morphine and its metabolites, and it is now clear that polymorphisms in *ABCC4* rs2274407, rs3742106, rs868853 and rs9561778 have a significant effect on plasma levels, drug efficacy and disease susceptibility (Anderson et al., 2006; Low et al., 2009; Venkatasubramanian et al., 2014; Tanaka et al., 2015; Sánchez-Martín et al., 2016; Chidambaran et al., 2017; Che et al., 2018). Similarly, *ABCC5* variants (rs562 and rs3749438) also have been reported to be associated with severe irinotecan-induced toxicity and its plasma concentration (Chen et al., 2015; Teft et al., 2015).

The pregnane X receptor (PXR, *NR1I2*), a prototypical member of the nuclear receptor superfamily, can be activated by a range of steroids or exogenous drugs to regulate the transcription of target genes, and plays an important role in the regulation of the environmental homeostasis and pathophysiological processes (Rogers et al., 2021). Atorvastatin acts as a ligand for PXR and activates transcription of target genes, including *CYP3A4, CYP3A5, SLC O 1B1* and *ABCB1* (Marino et al., 2011; Hoffart et al., 2012), thereby affecting the metabolic process of the drug. Many studies have reported that polymorphisms in *NR1I2* (rs6785049 and rs1523127) impact the pharmacokinetics of a variety of drugs, including immunosuppressive agents (Fanta et al., 2010; Mbatchi et al., 2017), antifungal agents (Zeng et al., 2020), antineoplastic agents (Liu et al., 2017) and anti-AIDS agents (Swart et al., 2012).

Accordingly, we examined the effects of reported polymorphisms in metabolic enzymes (*CYP3A4* and *CYP3A5*) and transporters (*SLC O 1B1, ABCB1* and *ABCG2*) associated with atorvastatin efficacy, as well as other drug transport-related gene (*ABCC3, ABCC4, ABCC5* and *NR1I2*) variants on plasma concentrations of atorvastatin and its metabolites in patients with CKD. The study of individual variation in plasma concentrations of atorvastatin and its metabolites at the genetic level is helpful to predict the efficacy and toxicity of atorvastatin in CKD patients accurately, and has practical guiding significance for clinical individualized drug application.

## 2 Materials and methods

### 2.1 Clinical pharmacogenetic study

#### 2.1.1 Study population

A prospective study was performed with Chinese Han chronic kidney disease patients recruited from both out-patients and in-patients of the Nephrology Departments of the First and Second Affiliated Hospitals of Shantou University Medical College, from May 2014 to September 2019 (Chinese Clinical Trial Registry No. ChiCTR2000041391). According to the inclusion and exclusion criteria (Supplementary Table S1), there were 354 eligible CKD patients, 142 patients were excluded due to missing data, a total of 212 CKD patients participated in our study. All patients were prescribed atorvastatin (Pfizer, NY, America) 20 mg/day for 6 weeks. Overnight fasting blood samples were collected from patients to measure biochemical parameters and drug levels. Baseline parameters were measured the morning of the day of treatment initiation, and final parameters were measured the day the 6-week treatment was completed. Demographic and clinical information, including age, sex, body, and medical histories were recorded. Estimated glomerular filtration rate (eGFR) was calculated according to the Chronic Kidney Disease Epidemiology Collaboration (CKD-EPI) equation (Levey et al., 2009). Experimental subjects were not randomized into groups and the experimenters were not blinded because these were deemed inappropriate for the design of this study. This study was approved by the ethics committees of the First and Second Affiliated Hospitals of Shantou University Medical College. All enrolled patients were informed of the purpose and other matters of the study, understood and signed the informed consent form.

#### 2.1.2 Genotyping

DNA was isolated from EDTA-coated whole blood tubes, following patient blood collection, using a TIANamp blood DNA kit (TIANGEN, Beijing, China). The concentration and purity of extracted DNA following the manufacturer’s protocols were determined with a Nanodrop 2000.

Genotyping was performed using TaqMan^®^ Real Time Polymerase Chain Reaction (PCR) allelic discrimination assays, with a Drug Metabolism Enzyme or predesigned probe and primer (Applied Biosystems, CA, United States), according to the manufacturer’s instructions. TaqMan drug metabolism enzyme genotyping assays (for *ABCB1* rs1128503, rs2032582, rs1045642, *ABCG2* rs2231142, *SLC O 1B1* rs2306283, rs4149056, *CYP3A5* rs776746, *CYP3A4* rs2242480, *ABCC4* rs3742106, rs2274407 and *NR1I2* rs1523127) used different PCR conditions from the predesigned TaqMan SNP genotyping assays (for *CYP3A4* rs4646437, *ABCC4* rs9561778, rs868853, *ABCC3* rs4793665, *ABCC5* rs562, rs3749438 and *NR1I2* rs6785049). Conditions for the former were as follows: 95°C for 10 min, followed by 50 cycles of 95°C for 15 s and 60°C for 90 s. For the latter, 40 cycles of a 1 min annealing/1 min extension were used. Hardy-Weinberg equilibrium tests were performed using chi square tests, where the allele frequencies of the study population were consistent with the law of genetic equilibrium, as indicated by a *p*-value >0.05.

#### 2.1.3 Determination of atorvastatin and its metabolite concentrations

The concentrations of atorvastatin and its metabolites (2-AT, 4-AT, ATL, 2-ATL and 4-ATL) were quantified in EDTA plasma samples from the 6 week, when the concentration of atorvastatin and its metabolites were considered to remain at a steady-state level, by using an UPLC-MS/MS assay with a lower limit of quantification of 5 ng/mL. Blood samples from patients were mixed with 300ul internal standard solution (100 ug/ml methaqualone) in 2 ml polypropylene tubes, and the mixtures were vortex-mixed for 30 s then cenrtifuged at 14,000 rpm for 10 min at 4°C. A 3 μL aliquot was injected into a 2.7 µm Poroshell 120 EC-C18 column (4.6 × 100 mm, Agilent Technologies, Santa Clara, CA United States of America), and analytes were separated using gradient elution with 0.1% formic acid in water and methanol at a flow rate of 0.3 mL/min. Analyte detection was *via* multiple reaction monitoring (Sciex triple quadrupole 6,500 QTRAP mass spectrometer with a Turbo V electrospray source, AB Sciex, United States) using transitions of (M + H^+^) m/z): AT 559.3→440.2, 2-AT 575.2→440.2 4-AT 575.2→440.2, ATL 541.3→448.3, 2-ATL 557.2→448.2, 4-ATL 557.2→448.2, and methaqualone 251.2→132.2.

### 2.2 Plasmid construction and luciferase assay

Human embryonic kidney 293T cells (HEK293T) and human hepatocellular carcinoma cells (HepG2) were cultured at 37°C with 5% CO_2_ and 95% humidity in Dulbecco’s modified Eagle’s medium (Gibco, United States) supplemented with 10% fetal bovine serum, 100 U/mL penicillin G sodium, and 100 μg/mL streptomycin sulfate (Gibco).

The 1740 bp 3′UTR of *ABCC4* (NM_005845), with incorporated terminal Xba1 restriction sites, was amplified human genomic DNA with PCR primers. The fragment was then cloned into the luciferase reporter plasmid pGL3-promoter, and creation of the rs3742106 (T/G) polymorphism was achieved by site-directed mutagenesis. Similarly, the promoter of *ABCC4* gene was amplified with the primers 5′-TTT​CTC​TAT​CGA​TAG​GTA​CCT​AGG​ATT​ATA​GGC​GTG​AGC​C-3' (forward) and 5′-CTT​AGA​TCG​CAG​ATC​TCG​AGG​CTG​GGG​CTC​CGG​CCG​CCA​CGC​C-3' (reverse). The products were digested with restriction endonucleases KpnI and XhoI, and then were cloned into pGL3-Basic vector, and the rs868853 (G/A) polymorphism was generated by using PCR-based site-directed mutagenesis. The recombinant plasmids were validated by PCR, endonuclease digestion, and DNA sequencing.

Plasmid DNA was isolated with a PureLink HiPure Plasmid Midiprep kit (Invitrogen, CA, United States), and supercoiled plasmid DNA was transfected into HEK293T and HepG2 cells using Lipofectamine 2,000 transfection reagent (Invitrogen) according to the manufacturer’s protocol. The cells were plated in 12-well plates 1 day before transfection at a density of 1 × 10^5^ cells per well, and then were transfected with 2 μg of the reporter construct, 200 ng Renilla plasmid DNA and 2 μL Lipofectamine 2,000 after reaching 70%–80% confluence. After transfection for 24 h, cells were lysed for sequential measurement with the dual luciferase assay system (Promega, United States) in the ultra-sensitive GloMax Navigator Detection System instrument (Promega). Luciferase results are expressed as the ratio of firefly luciferase activity to Renilla luciferase activity from triplicate transfections.

### 2.3 Statistical analysis

Categorical data are presented as percentages, and continuous variables complied with normal distribution were expressed as mean ± standard deviation (M ± SD). The Shapiro-Wilk test was used for normality testing and non-normal distributed variables are presented as the median ± interquartile range (M ± Q). The correlation between plasma concentrations of atorvastatin and its metabolites were analyzed by Spearman correlation analysis as these variables were skewed even with a log transformation.

Considering that the lactonization and hydrolysis of atorvastatin and its metabolites are reversible processes, the lactone metabolisms and their corresponding hydroxyl acids were analyzed as a whole (AT + ATL, 2-AT+2-ATL, 4-AT+4-ATL). The sum of the active components of atorvastatin (AT+2-AT+4-AT) was also analyzed. Plasma concentrations of atorvastatin and its metabolites below the lower limit of detection were excluded. Outlier values (mean ± 3*SD) suggestive of errors in sampling procedure, technical measurements or data manipulation were excluded from the analysis.

Ancestral alleles from the Ensembl Genome database were defined as wild-type alleles, and the association between the genetic polymorphisms and the plasma concentration of atorvastatin and its metabolites were assessed in different genetic models (general, dominant, recessive models) by Mann–Whitney *U* or Kruskal–Wallis *H* tests. The addition concentration of atorvastatin lactone metabolites and their corresponding hydroxyl acids was log-transformed and then analyzed with clinical variables in univariate analyses. Any independent variables with a *p*-value of <0.1 in the univariate analysis were entered into a model of multivariable regression analysis to assess the influences of clinical variables on drug concentrations using the stepwise method. Finally, adjusting for gender, age, smoking and alcohol consumption, multivariable linear regression methods were used to evaluate the effects of genetic variation and baseline biochemical indices on plasma concentrations of atorvastatin and its metabolites. Statistical significance was defined having a two-sided *p*-value <0.05. Statistical analyses were performed using SPSS software (version 23, IBM, United States), and box and whisker plotting was performed using GraphPad Prism software (version 8.0.2.263, GraphPad, United States).

## 3 Results

### 3.1 Clinical characteristics of the study population

A total of 212 CKD patients were included in this study. The baseline clinical information of the patients is shown in Table 1. The average age of the patients was about 56 years old, and the male to female ratio was 1.2:1, 79.5% of patients had hypertensive disease, 42.6% had diabetes mellitus and 29.1% had cardiovascular disease. After 6 weeks of atorvastatin treatment, lipid levels of patients with CKD significantly improved.

**TABLE 1:** Clinical and demographic characteristics of the study population for atorvastatin treatment (20 mg/day/6 weeks).

Characteristics	Total study population (n = 212)
Age (years)	56.4 ± 10.9
Males, N (%)	116 (54.7)
Body mass index (kg/m^2^)	23.2 ± 4.64
Systolic blood pressure (mmHg)	142.0 ± 30.0
Diastolic blood pressure (mmHg)	85.5 ± 12.0
Hypertension, N (%)	167 (79.5)
Diabetes, N (%)	87 (42.6)
Cardiovascular disease, N (%)	53 (29.1)
Smoking, N (%)	61 (28.9)
Alcohol Consumption, N (%)	14 (6.6)

Lipid level	Baseline	Post-Treatment	Change	*p*-value
TC (mmol/L)	5.89 ± 1.86	4.06 ± 1.09	−1.77 ± 1.56	<**0.001**
TG (mmol/L)	2.08 ± 1.72	1.54 ± 1.01	−0.32 ± 0.81	<**0.001**
HDL-C (mmol/L)	1.26 ± 0.41	1.23 ± 0.38	−0.04 ± 0.29	**0.026**
LDL-C (mmol/L)	3.86 ± 1.29	2.51 ± 0.96	−1.32 ± 1.06	<**0.001**
ApoAI (g/L)	1.40 ± 0.33	1.48 ± 0.37	0.04 ± 0.26	**0.033**
ApoB100 (g/L)	1.09 ± 0.34	0.74 ± 0.30	−0.33 ± 0.3	<**0.001**
LP(a) (mg/L)	292.75 ± 255.75	295.46 ± 279.41	−2.7 ± 89.45	0.831

Abbreviations: TC, total cholesterol; TG, HDL-C, High Density Lipoprotein-Cholesterol; LDL-C, Low Density Lipoprotein-Cholesterol; ApoAI, Apolipoprotein AI; ApoB100, Apolipoprotein B100; LP(a), Lipoprotein (a).

Genotyped variants and their distribution in the population studied are shown in Table 2. All genotypes tested were in Hardy-Weinberg equilibrium with the exception of *CYP3A5* rs776746 (*p* = 0.003) and *ABCB1* rs2032582 (*p* = 0.027), and the minor allele frequency (MAF) was close to the MAF of the Southern Chinese Han Chinese in the 1,000 Genomes database (https://www.ncbi.nlm.nih.gov/snp/) except for rs776746 and rs2032582, and are given in Supplementary Table S2.

**TABLE 2:** Genotyped variants and their distribution in the studied population (n = 210).

Gene	SNPs	CHR	Location	Genotype	N (%)	MAF
*CYP3A4*	rs2242480 (intron)	7	99763843	TT	21 (10.0)	T = 0.312
				CT	89 (42.4)	
				CC	100 (47.6)	
*CYP3A4*	rs4646437 (intron)	7	99767460	AA	12 (5.7)	A = 0.193
				AG	57 (27.1)	
				GG	141 (67.1)	
*CYP3A5*	rs776746 (intron)	7	99672916	CC	69 (32.9)	T = 0.474
				CT	83 (39.5)	
				TT	58 (27.6)	
*SLC O 1B1*	rs4149056 (missense variant)	12	21178615	CC	4 (1.9)	C = 0.114
				CT	40 (19.0)	
				TT	166 (79.0)	
*SLC O 1B1*	rs2306283 (missense variant)	12	21176804	AA	10 (4.8)	A = 0.202
				AG	65 (31.0)	
				GG	135 (64.3)	
*ABCB1*	rs1128503 (synonymous variant)	7	87550285	AA	98 (46.7)	G = 0.31
				AG	94 (44.8)	
				GG	18 (8.6)	
*ABCB1*	rs1045642 (missense variant)	7	87509329	AA	31 (14.8)	A = 0.412
				AG	111 (52.9)	
				GG	68 (32.4)	
*ABCB1*	rs2032582 (missense variant)	7	87531302	CC	49 (23.3)	T = 0.1
				CA/CT	106 (50.5)	
				AA/TT/AT	55 (26.2)	
*ABCG2*	rs2231142 (missense variant)	4	88131171	GG	78 (37.1)	T = 0.369
				GT	109 (51.9)	
				TT	23 (11.0)	
*ABCC3*	rs4793665 (2 kb upstream)	17	50634726	CC	5 (2.4)	C = 0.131
				CT	45 (21.4)	
				TT	160 (76.2)	
*ABCC4*	rs3742106 (3′UTR)	13	95021537	AA	43 (20.5)	A = 0.486
				AC	118 (56.2)	
				CC	49 (23.3)	
*ABCC4*	rs9561778 (intron)	13	95061461	GG	92 (43.8)	T = 0.331
				GT	97 (46.2)	
				TT	21 (10.0)	
*ABCC4*	rs2274407 (missense variant)	13	95206781	AA	5 (2.4)	A = 0.152
				AC	54 (25.7)	
				CC	151 (71.9)	
*ABCC4*	rs868853 (2 kb upstream)	13	95302822	CC	5 (2.4)	C = 0.129
				CT	44 (21.0)	
				TT	161 (76.7)	
*ABCC5*	rs562 (3′UTR)	3	183920057	CC	46 (21.9)	C = 0.493
				CT	115 (54.8)	
				TT	49 (23.3)	
*ABCC5*	rs3749438 (intron)	3	183987396	AA	40 (19.0)	A = 0.436
				AG	103 (49.0)	
				GG	67 (31.9)	
*NR1I2*	rs6785049 (intron)	3	119814886	AA	41 (19.5)	A = 0.412
				AG	91 (43.3)	
				GG	78 (37.1)	
*NR1I2*	rs1523127 (5′UTR)	3	119782192	AA	121 (57.6)	C = 0.229
				AC	82 (39.0)	
				CC	7 (3.3)	

Notes: 2 blood samples were missing.

Abbreviations: SNP, single nucleotide polymorphism; CHR, chromosome; MAF, minor allele frequency.

### 3.2 Atorvastatin and its metabolite concentrations

**FIGURE 2:**
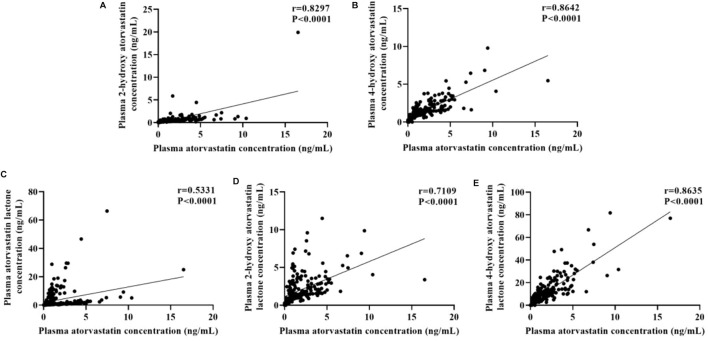
Correlations between plasma concentration of atorvastatin and its metabolites: **(A)** 2-hydroxy atorvastatin; **(B)** 4-hydroxy atorvastatin; **(C)** atorvastatin lactone; **(D)** 2-hydroxy atorvastatin lactone; **(E)** 4-hydroxy atorvastatin lactone.

The plasma concentrations of atorvastatin and its metabolites varied widely among individuals, with patient plasma concentrations of atorvastatin ranging from 0.097 to 16.520 ng/mL, 2-hydroxy atorvastatin from 0.062 to 19.900 ng/mL, 4-hydroxy atorvastatin from 0.179 to 9.785 ng/mL, atorvastatin lactone from 0.064 to 66.390 ng/mL, 2-hydroxy atorvastatin lactone concentrations ranged from 0.196 to 11.510 ng/mL and 4-hydroxy atorvastatin lactone concentrations ranged from 0.398 to 81.700 ng/mL. Spearman’s analysis of the correlation between atorvastatin and its metabolites showed that the concentrations of atorvastatin metabolites significantly correlated with the parent drug (r > 0.5, *p* < 0.0001) (Figure 2).

### 3.3 Association between gene polymorphisms with plasma concentrations of atorvastatin and its metabolites

First, the effect of baseline clinical characteristics of plasma concentrations of atorvastatin and its metabolites was evaluated by univariate and multivariate analyses. A high Apolipoprotein AI (apoAI) level was correlated with higher plasma levels of AT + ATL (β = 0.421, *p* = 0.003), and baseline Lipoprotein a (LPa) (β = 2.44E-04, *p* = 0.037) and glucose (β = −0.019, *p* = 0.027) had an independent influence on the concentration of 2-AT+2-ATL. In terms of the plasma level of AT+2-AT+4-AT, it depended in part on levels of apoAI (β = 0.374, *p* < 0.001), ALB (β = -0.013, *p* = 0.012), glucose (β = −0.023, *p* = 0.012), eGFR (β = -0.004, *p* = 0.031) and alcohol consumption (β = −0.303, *p* = 0.015), indicating exposure levels of atorvastatin and its metabolites may be affected by hepatic and renal function (Supplementary Table S3 **)**.

The effect of genetic polymorphisms on plasma concentration of atorvastatin and its metabolites was analyzed in different genetic models (Supplementary Tables S4A–C). In the general model, three SNPs of *ABCC4* were significantly associated with the concentration of atorvastatin and its metabolites, showing that plasma 2-AT+2-ATL and AT+2-AT+4-AT levels were lower in individuals with the rs3742106 homozygous variant allele (CC) than the wild-type allele carriers (approximately reduced by 42% in both, *p* < 0.05, Table 4, Table 5), but the plasma concentration of 2-AT+2-ATL significantly increased in patients with the rs868853 heterozygous genotype (CT) when compared with the TT genotype (*p* = 0.015, CT vs TT) (Table 4). After correcting *p*-values with the Bonferroni-Dunn test, no association between rs9561778 polymorphism and plasma AT + ATL levels was observed (Table 3).

**TABLE 3:** Association between SNPs and plasma concentrations of AT + ATL.

Gene	SNP	Model	Genotypes	Cases	AT + ATL, μmol/L	*p*-value
					M ± Q	
*ABCC4*	rs9561778	General	GG	88	0.0075 ± 0.0105	Ref
			GT	94	0.0051 ± 0.0095	**0.05**
			TT	20	0.0080 ± 0.0150	0.458
*ABCC4*	rs9561778	Dominant	GG	88	0.0075 ± 0.0105	**0.039**
			GT + TT	114	0.0053 ± 0.0098	
*ABCC4*	rs3742106	Dominant	AA	42	0.0078 ± 0.0174	**0.034**
			AC + CC	160	0.0059 ± 0.0094	
*ABCB1*	rs1128503	Recessive	AA	95	0.0084 ± 0.0111	**0.047**
			AG + GG	107	0.0050 ± 0.0090	

Notes: bold fonts indicates *p* < 0.05.

In the dominant model, patients carrying the variant rs3742106 allele had lower levels of 2-AT+2-ATL than homozygous wild-type allele carriers (*p* = 0.015, AA vs AC + CC) (Table 4), and the homozygous wild-type rs9561778 allele carriers showed higher plasma concentrations of AT + ATL (*p* = 0.029, GG vs GT + TT). Interestingly, subjects with one or two copies of the variant *ABCC4* rs868853 or *NR1Ⅰ2* rs6785049 allele had increased plasma concentrations of 2-AT+2-ATL (*p* = 0.017, TT vs CT + TT; *p* = 0.048, GG vs AG + AA) (Table 4). For the *CYP3A4* rs464637 polymorphism, although the plasma concentration of 4-AT+4-ATL was distributed differently in carriers of different genotypes, there were only 10 cases of the AA genotype in CKD patients, which may reduce the statistical power.

**TABLE 4:** Association analysis between SNP and plasma concentration of 2-AT+2-ATL.

Gene	SNP	Model	Genotypes	Cases	2-AT+2-ATL,μmol/L	*p*-value
					M ± Q	
*ABCC4*	rs3742106	General	AA	40	0.0050 ± 0.0049	Ref
			AC	111	0.0039 ± 0.0042	0.158
			CC	48	0.0029 ± 0.0040	**0.017**
*ABCC4*	rs3742106	Dominant	AA	40	0.0050 ± 0.0049	**0.015**
			AC + CC	159	0.0036 ± 0.0044	
*ABCC4*	rs3742106	Recessive	CC	48	0.0029 ± 0.0040	**0.046**
			AC + AA	151	0.0041 ± 0.0041	
*ABCC4*	rs868853	General	TT	155	0.0036 ± 0.0042	Ref
			CT	39	0.0056 ± 0.0056	**0.015**
			CC	5	0.0026 ± 0.0055	0.219
*ABCC4*	rs868853	Dominant	TT	155	0.0036 ± 0.0042	**0.017**
			CT + CC	44	0.0050 ± 0.0057	
*NR1I2*	rs6785049	Dominant	GG	75	0.0035 ± 0.0045	**0.048**
			AG + AA	124	0.0039 ± 0.0043	
*CYP3A5*	rs776746	Recessive	CC	66	0.0044 ± 0.0054	**0.049**
			CT + TT	133	0.0036 ± 0.0042	

Notes: bold fonts indicates *p* < 0.05.

In the recessive model, the wild-type rs1128503 allele carriers had lower AT + ATL levels (*p* = 0.047, AA vs AG + GG) (Table 3), but plasma levels of 2-AT+2-ATL and AT+2-AT+4-AT decreased in individuals with the homozygous rs3742106 variant than wild-type allele carriers (*p* = 0.046; *p* = 0.008; CC vs AC + AA) (Table 4, Table 5). There were no significant associations between these polymorphisms and the plasma concentrations of 4-AT+4-ATL (Supplementary Table S4).

**TABLE 5:** Association analysis between genetic variants and plasma concentration of AT+2-AT+4-AT.

Gene	SNP	Model	Genotypes	Cases	AT+2-AT+4-AT,μmol/L	*p*-value
					M ± Q	
*ABCC4*	rs3742106	General	AA	42	0.0065 ± 0.0069	Ref
			AC	114	0.0053 ± 0.0074	0.377
			CC	49	0.0038 ± 0.0057	**0.027**
*ABCC4*	rs3742106	Recessive	CC	49	0.0038 ± 0.0057	**0.008**
			AC + AA	156	0.0056 ± 0.0074	

Notes: bold fonts indicates *p* < 0.05.

**FIGURE 3:**
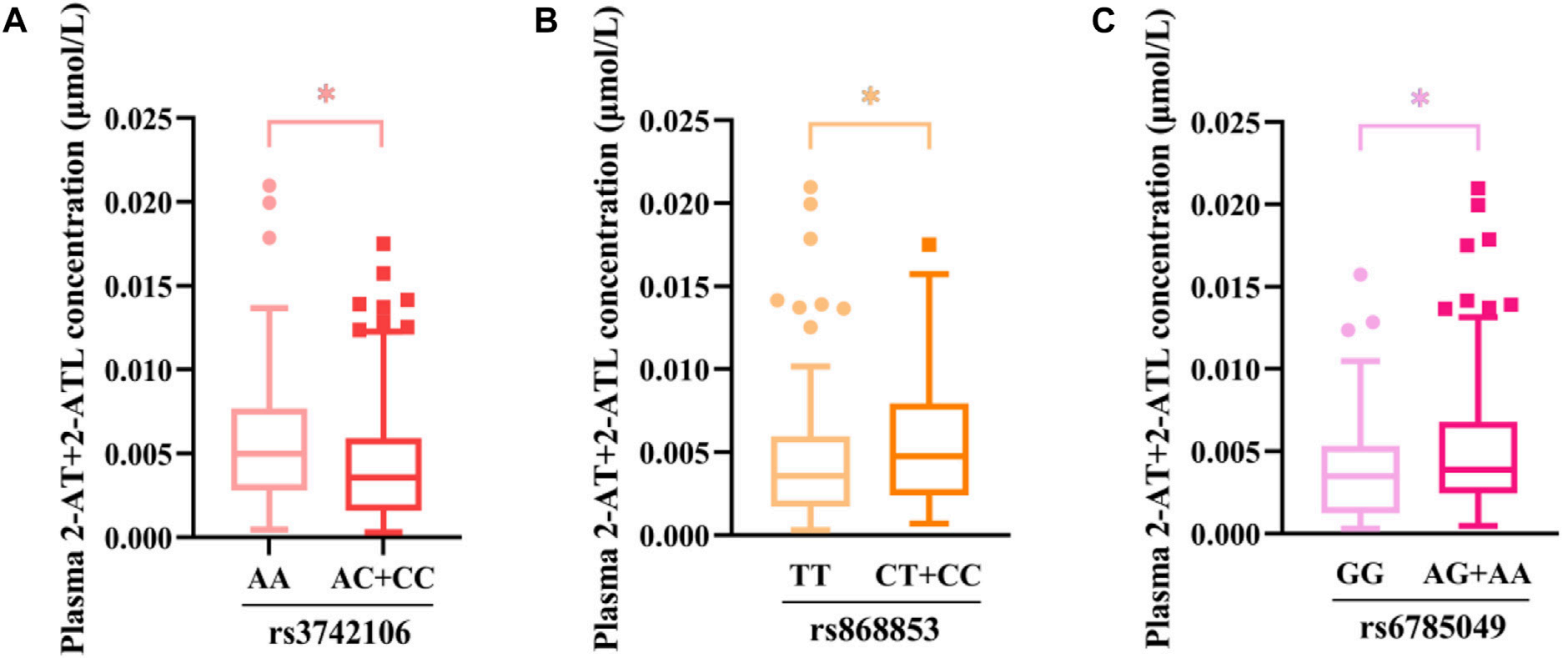
Association between genetic variants and the plasma concentration of 2-AT+2-ATL as determined using the Mann–Whitney *U* test. *p < 0.05. **(A)** rs3742106; **(B)**rs868853; **(C)** rs6785049.

Furthermore, multivariate linear regression analysis showed that the rs3742106 and rs868853 variants of *ABCC4* and the *NR1Ⅰ2* rs6785049 polymorphism were predictors of plasma 2-AT+2-ATL levels after adjustment for gender, age, smoking and alcohol consumption. In addition, baseline levels of lipoprotein (a) and glucose were correlated to the plasma 2-AT+2-ATL concentration, which together explained 14% of the variance in the plasma 2-AT+2-ATL concentration (Table 6; Figure 3). The *ABCC4* rs3742106CC genotype was a predictor of lower AT+2-AT+4-AT levels (β = −0.212, *p* = 0.028), and explained 19% of the variance in the plasma levels of this component with other significantly non-genetic clinical factors (Table 6).

**TABLE 6:** Plasma atorvastatin and its metabolite concentrations-linear regression model coefficients.

Variable	Effect (β)	*p*-value	*R* ^2^
2-AT+2-ATL			
Glucose	−0.019	**0.025**	
LP(a)	2.00E-04	0.083	
*ABCC4* rs3742106	−0.162	**0.028**	
*ABCC4* rs868853	0.177	**0.011**	
*NR1I2* rs6785049	0.123	**0.044**	0.14
AT+2-AT+4-AT			
ApoAⅠ	0.352	**0.001**	
ALB	−0.013	**0.006**	
Glucose	−0.021	**0.026**	
eGFR	−0.004	**0.016**	
*ABCC4* rs3742106CC	−0.212	**0.028**	
*ABCC4* rs3742106AC	−0.029	0.723	0.19

Notes: bold fonts indicates *p* < 0.05.

Adjusted for gender, age, smoking and alcohol consumption.

LP(a), lipoprotein (a); ApoAⅠ, Apolipoprotein AI; ALB, albumin; eGFR, estimated glomerular filtration rate.

### 3.4 Functional effects of genetic variants *in vitro*

**FIGURE 4:**
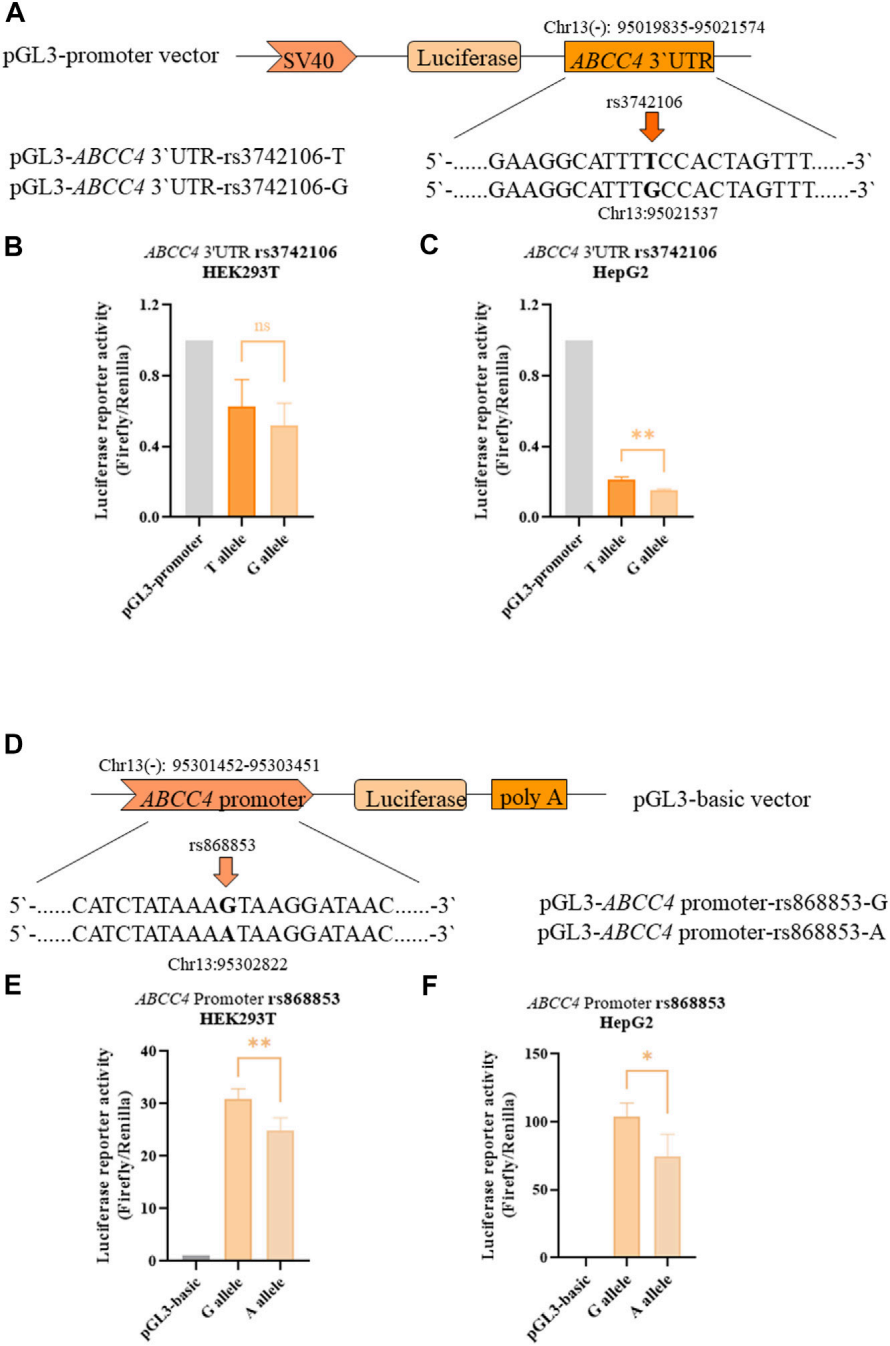
*ABCC4* rs3742106 and rs868853 affect luciferase reporter activity. **(A)** Schematic representation of the *ABCC4* 3′UTR expression plasmid and the position of corresponding mutant sites. Firefly luciferase constructs contained the SV40 promoter, luciferase coding region and a fragment of the *ABCC4* 3′UTR. The rs3742106 SNP polymorphism site is indicated by the arrow. Plasmid constructs were transfected into HEK293T **(B)** and HepG2 cells **(C)**. **(D)** Schematic representation of the *ABCC4* promoter expression plasmid and the position of the corresponding mutant sites. Firefly luciferase constructs contained 2 kb fragment of the *ABCC4* promoter, luciferase coding region and SV40 poly **(A)**. The rs868853SNP polymorphism site is indicated by the arrow. Plasmid constructs were transfected into HEK293T **(E)** and HepG2 cells **(F)**. Data represent the mean values of four independent experiments ±SD. Each experiment was conducted in triplicate. Ns indicates statistically non-significant; *p < 0.05, **p < 0.01 were considered as significant.

The clinical trial showed significant associations between *ABCC4* and *NR1Ⅰ2* polymorphisms (rs868853, rs3742106 and rs6785049) with plasma levels of atorvastatin and its metabolites, so we further investigated the functional effects of these genetic variants *in vitro*. Since rs6785049 is located in intron of *NR1Ⅰ2*, we only studied *ABCC4* polymorphisms, in the promoter and 3′ untranslated (3′UTR) regions, using a luciferase assay *in vitro*. The luciferase constructs and information of the polymorphism sites are shown in Figures 4A,D. The constructs, which were comprised of the *ABCC4* sequence (promoter or 3′UTR region) and a luciferase reporter gene, were transfected into HEK293T and HepG2 cells. Functional studies showed variant alleles of rs868853-A had significantly lower luciferase activities than rs868853-G in both cell lines (HEK293T *p* = 0.0026, HeG2 *p* = 0.022), rs3742106-G had significantly lower luciferase activities than rs3742106-T in HepG2 cells lines (*p* = 0.0031), but not significantly in HEK293T (*p* = 0.31). (Figure 4).

## 4 Discussion

Individual differences in response to atorvastatin exist between patients. In this study, we investigated the association between genetic variants in drug metabolism- and transportation-related genes, and plasma concentrations of atorvastatin and its metabolites in patients with chronic kidney disease. Eighteen polymorphisms in 9 genes (*CYP3A4, CYP3A5, SLC O 1B1, ABCB1, ABCG2, ABCC3, ABCC4, ABCC5* and *NR1I2*) were identified and displayed non-significant deviation from Hardy-Weinberg equilibrium, except for rs776746 and rs2032582, indicating that the study population is representative. However, the reason why two SNPs were not in equilibrium in our population is unclear.

DeGorter et al. demonstrated a high, 45-fold inter-individual variability in circulating atorvastatin levels following treatment with the same dose (DeGorter et al., 2013). Similarly, we found large individual differences in plasma concentrations of atorvastatin and its metabolites, especially for its lactone metabolites (over 80-fold). Considering that lactonization and hydrolysis of atorvastatin is reversible with a change in pH (Jemal and Xia, 2000), the lactone metabolites and their corresponding hydroxyl acids were analyzed as a whole, avoiding conversion that might be affected by environmental factors in the disposal of blood samples, and correlated with genetic polymorphisms. The results from univariate and multivariate analyses showed that the *ABCC4* rs3742106 polymorphism was associated with plasma concentrations of AT and its metabolite, indicating patients carrying the wild-type allele (C) have higher 2-AT+2-ATL and AT+2-AT+4-AT levels than variant allele (A) carriers. We further show rs3742106, located in the region of *ABCC4* encoding the 3′UTR, is an independent factor affecting plasma concentrations of AT and its metabolites.

*ABCC4* is located on chromosome 13q32.1, contains 31 exons and encodes the multidrug resistance-associated protein MRP4, which is localized in the basolateral membrane of cells in the liver, but is more highly expressed in the parietal membrane of renal tubular cells in the kidney (Russel et al., 2008). *ABCC4* polymorphisms have mostly been reported to correlate with tumor prognosis and the efficacy of anti-HIV drugs. Anderson et al. reported that plasma lamivudine triphosphate concentrations were 20% higher in patients carrying the rs3742106 variant allele C than in carriers with the homozygous AA genotype (Anderson et al., 2006). Similarly, Rungtivasuwan et al. also found the plasma concentrations of tenofovir were 30% higher in patients carrying variant allele than in the AA genotype carriers infected with HIV (Rungtivasuwan et al., 2015), which is opposite to our findings. However, these seemingly paradoxical results can be explained on the basis of the metabolic characteristics of the drug and the localization of MRP4. Anti-HIV drugs are mainly excreted by the kidneys, thus the level of MRP4 efflux transport in the parietal membrane of renal tubular cells can directly influence the plasma level of anti-HIV drugs. Thus, rs3742106C may reduce MRP4 protein expression, resulting in reduced efflux of drugs in renal tubular cells (Anderson et al., 2006), allowing an increase in drug plasma concentrations. In contrast, atorvastatin is metabolized in the liver and mostly eliminated by the bile, with only about 1% excreted by the kidneys (Lennernäs, 2003). Combining the correlation between rs3742106 and plasma concentrations of AT and its metabolites with the reporter gene results, it is hypothesized that rs3742106C also decreases hepatic MPR4 protein expression, leading to a decrease in drug excretion from hepatocytes into the hepatic sinusoids and into the circulation, resulting in a decrease in plasma levels of AT and its metabolites. These results further validate Anderson’s first hypothetical description of rs3742106 affecting the expression of *ABCC4*.

Variant allele carriers of rs868853(C), which is located in the *ABCC4* promoter, exhibited higher 2-AT+2-ATL levels than homozygous wild-type carriers (TT), and rs868853 was found to be an independent influence on plasma concentrations of 2-AT+2-ATL after adjusting for non-genetic factors by multiple linear regression. Palikhe et al. showed asthma patients with the rs868853G allele had higher concentrations of asthma severity markers in blood and urine, and that the rs868853G SNP increased *ABCC4* promoter transcriptional activity, suggesting that rs868853C may be associated with the transport of metabolites from immune cells (Palikhe et al., 2017). Additionally, carriers of the rs868853T allele were found to have a lower susceptibility to Kawasaki disease in a southern Chinese population (Che et al., 2018). However, there has been no reported on the association between this SNP and the pharmacokinetics or pharmacodynamics of drugs. To the best of our knowledge, this is the first report on the influence of rs868853 on plasma concentrations of AT and its metabolites. Interestingly, luciferase assays demonstrated the *ABCC4* rs868853G allele enhanced transcription compared with the rs868853A allele, which is consistent with Palikhe’s results. Taken together, the rs868853(C/G) variant allele may enhance the transcriptional activity of *ABCC4*, resulting in enhanced expression of MRP4, leading to increased efflux of AT and its metabolites from the basal membrane of the hepatocyte into the circulation.

It was reported that rs9561778, located in an intron of *ABCC4*, was associated with cyclophosphamide-induced adverse effects (gastrointestinal toxicity and leukopenia or neutropenia) in patients with breast cancer, and FASTSNP suggested that rs9561778 may be located within transcription factor binding sites or intron enhancer sequences, and is a pathogenic variant affecting gene expression (Low et al., 2009). In our study, we found patients carrying the rs9561778 (T) variant allele had decreased concentrations of AT + ATL compared to homozygous wild-type (GG) carriers, but there was no statistical difference in our multiple linear regression analysis, suggesting that rs9561778 is not an independent factor affecting plasma concentrations of AT and its metabolites. Therefore, the association between the rs9561778 and plasma concentration of AT and its metabolites still needs to be further explored. Our results explain the clinical and cellular evidence that the polymorphisms of *ABCC4* are associated with the circulating levels of AT and its metabolites, which changed the expression of the transporter encoded by *ABCC4*.

The pregnane X receptor (PXR), is encoded by *NR1I2* on chromosome 3q12/13.3, and contains nine exons, but only exons two to nine are involved in encoding protein (Zhang et al., 2001). PXR is widely expressed in a variety of tissues, mainly in the liver and intestine (Lamba et al., 2005). Genetic polymorphisms in PXR may explain the variation in the expression of the target genes (Lamba et al., 2005), which may affect the pharmacokinetics or pharmacodynamics of atorvastatin. Several studies have demonstrated a relationship between rs6785049 and the plasma concentrations of various drugs (Miura et al., 2008; Mbatchi et al., 2017; Zeng et al., 2020). When we considered polymorphisms in *NR1I2*, we found rs6785049A was associated with increased concentration of 2-AT+2-ATL. Additionally, active metabolites (2-AT and 4-AT) of atorvastatin also are ligands of PXR, and 4-AT reduced induction of genes regulated by PXR compared to 2-AT and AT *in vitro*, which may be due to the weaker ability of the 4-AT to induce expression of target genes as the result of the retention of co-repressors during ligand activation (Hoffart et al., 2012). We show a genetic variant in PXR is associated with the plasma concentration of 2-AT+2-ATL, which may partly be explained in part by the metabolism of atorvastatin, i.e. since active metabolites are produced by CYP3A, acting as ligand to activate PXR regulates *CYP3A4/CYP3A5*, thereby producing more hydroxylated metabolites especially of 2-AT. Moreover, it has been reported that individuals with the GG genotype have twice the expression levels of *CYP3A* in enterocytes, than rs6785049A carriers, after the activation of PXR (Zhang et al., 2001). Taken together, these results imply that multiple pathways act together to cause differences in plasma 2-AT+2-ATL concentrations in patients carrying different genotypes.

*CYP3A5* is another atorvastatin metabolizing enzyme, and its genetic polymorphisms have an important impact on the distribution of drugs (Zubiaur et al., 2021), although it has been found that *CYP3A5* contributes less than 15% to atorvastatin metabolism *in vitro* (Park et al., 2008). CYP3A5*3 (rs776746C) is the major *CYP3A5* allele that found in all ethnic groups (Zhou et al., 2017) and is functionally deficient (Kuehl et al., 2001). In our study, we found patients with the homozygous CC variant genotype have higher levels of 2-AT+2-ATL compared to the wild-type allele carriers. A previous study by Kim et al. found that after a single dose of simvastatin, the AUC_0-12_ was 3.3-fold higher in healthy individuals with the CC genotype than in those with the TT genotype (Kim et al., 2007). On the contrary, Zubiaur et al. indicated individuals carrying the C allele had lower plasma concentration of atorvastatin than the TT genotype carriers (Zubiaur et al., 2021). Significantly, the *CYP3A5* rs776746 polymorphism was not in equilibrium in our population. Thus, further association analysis between rs776746 and plasma concentrations of AT and its metabolites, in large representative groups, is needed to clarify the reason for the different conclusions.

The P-glycoprotein protein encoded by *ABCB1* plays an important role in the absorption of oral drugs in the intestine, and polymorphisms of this gene have been reported to be related to the lipid-lowering effect of atorvastatin (Thompson et al., 2005; Rebecchi et al., 2009; Hoenig et al., 2011; Kadam et al., 2016). Although rs1045642, located in exon 26 of *ABCB1*, does not change the expression of mRNA and protein, it may change of P-glycoprotein folding (Kimchi-Sarfaty et al., 2007). Non-etheless, there is a lack of studies into the effect of *ABCB1*-rs1045642 on the metabolism of patients to atorvastatin. *ABCB1* rs1045642AA was associated with higher plasma concentrations of AT + ATL in this study. Therefore, this association still needs to be further verified.

Although a number of polymorphisms have been suggested as candidates for the pharmacokinetic variability of atorvastatin, the present study is the first to indicate the involvement of *ABCC4*-rs3742106 and rs868853, as well as *NR1I2*-rs6785049. While the current study demonstrates the contribution of genetic polymorphisms to the plasma concentrations of atorvastatin and its metabolites, there are a number of limitations. First, the sample size of this study is limited, and of a single ethnic population. Secondly, although drugs that may interact with atorvastatin have been excluded from the inclusion criteria, patients may have taken other medications during the 6 weeks of statin use, which may have had an impact on atorvastatin, and some drugs may have undetected effects on the statin, so factors of drug combination should be carefully analyzed in future research. In addition, it has been reported that chronic kidney disease affects the pharmacokinetics of drugs cleared by non-renal mechanisms (Yeung et al., 2014), thus the results of this study are only applicable to the CKD population.

## 5 Conclusion

In conclusion, the genetic variants in drug metabolism- and transportation-related genes and their effects on plasma concentrations of atorvastatin and its metabolites were identified and characterized in CKD patients. The present findings suggest the CC genotype of *ABCC4*-rs3742106 is associated with decreased concentrations of AT and its metabolites, whereas patients carrying the *ABCC4*-rs868853 or *NR1I2*-rs6785049 variant had higher concentrations of 2-AT+2-ATL in plasma compared with homozygous wild-type carriers with CKD. In addition, we also found that two *ABCC4* SNPs may affect transcriptional activity, thereby affecting release of AT and its metabolites from hepatocytes into the circulation. These novel findings increase the panel of potential genetic biomarkers related to atorvastatin metabolism, and the results also help to improve the efficacy and toxicity prediction of atorvastatin in patients with CKD.

## Data Availability

The original contributions presented in the study are included in the article/Supplementary Materials, further inquiries can be directed to the corresponding authors.

## References

[B1] Abd T. T. Jacobson T. A. (2011). Statin-induced myopathy: A review and update. Expert Opin. Drug Saf. 10 (3), 373–387. 10.1517/14740338.2011.540568 21342078

[B2] Adams S. P. Tsang M. Wright J. M. (2015). Lipid-lowering efficacy of atorvastatin. Cochrane Database Syst. Rev. 2015 (3), Cd008226. 10.1002/14651858.CD008226.pub3 25760954 PMC6464917

[B3] Anderson P. L. Lamba J. Aquilante C. L. Schuetz E. Fletcher C. V. (2006). Pharmacogenetic characteristics of indinavir, zidovudine, and lamivudine therapy in HIV-infected adults: A pilot study. J. Acquir Immune Defic. Syndr. 42 (4), 441–449. 10.1097/01.qai.0000225013.53568.69 16791115

[B4] Arca M. Gaspardone A. (2007). Atorvastatin efficacy in the primary and secondary prevention of cardiovascular events. Drugs 67, 29–42. 10.2165/00003495-200767001-00004 17910519

[B5] Aviram M. Rosenblat M. Bisgaier C. L. Newton R. S. (1998). Atorvastatin and gemfibrozil metabolites, but not the parent drugs, are potent antioxidants against lipoprotein oxidation. Atherosclerosis 138 (2), 271–280. 10.1016/s0021-9150(98)00032-x 9690910

[B6] Birmingham B. K. Bujac S. R. Elsby R. Azumaya C. T. Wei C. Chen Y. (2015). Impact of ABCG2 and SLCO1B1 polymorphisms on pharmacokinetics of rosuvastatin, atorvastatin and simvastatin acid in caucasian and asian subjects: A class effect? Eur. J. Clin. Pharmacol. 71 (3), 341–355. 10.1007/s00228-014-1801-z 25673568

[B7] Che D. Pi L. Fang Z. Xu Y. Cai M. Fu L. (2018). ABCC4 variants modify susceptibility to Kawasaki disease in a southern Chinese population. Dis. Markers 2018, 8638096. 10.1155/2018/8638096 30363999 PMC6186368

[B8] Chen S. Villeneuve L. Jonker D. Couture F. Laverdière I. Cecchin E. (2015). ABCC5 and ABCG1 polymorphisms predict irinotecan-induced severe toxicity in metastatic colorectal cancer patients. Pharmacogenet Genomics 25 (12), 573–583. 10.1097/fpc.0000000000000168 26352872

[B9] Chidambaran V. Venkatasubramanian R. Zhang X. Martin L. J. Niu J. Mizuno T. (2017). ABCC3 genetic variants are associated with postoperative morphine-induced respiratory depression and morphine pharmacokinetics in children. Pharmacogenomics J. 17 (2), 162–169. 10.1038/tpj.2015.98 26810133 PMC4959996

[B10] Cooper-DeHoff R. M. Niemi M. Ramsey L. B. Luzum J. A. Tarkiainen E. K. Straka R. J. (2022). The clinical pharmacogenetics implementation Consortium guideline for SLCO1B1, ABCG2, and CYP2C9 genotypes and statin-associated musculoskeletal symptoms. Clin. Pharmacol. Ther. 111, 1007–1021. 10.1002/cpt.2557 35152405 PMC9035072

[B11] Cruz-Correa O. F. León-Cachón R. B. Barrera-Saldaña H. A. Soberón X. (2017). Prediction of atorvastatin plasmatic concentrations in healthy volunteers using integrated pharmacogenetics sequencing. Pharmacogenomics 18 (2), 121–131. 10.2217/pgs-2016-0072 27976987

[B12] DeGorter M. K. Tirona R. G. Schwarz U. I. Choi Y. H. Dresser G. K. Suskin N. (2013). Clinical and pharmacogenetic predictors of circulating atorvastatin and rosuvastatin concentrations in routine clinical care. Circ. Cardiovasc Genet. 6 (4), 400–408. 10.1161/circgenetics.113.000099 23876492 PMC3922121

[B13] Deng F. Tuomi S. K. Neuvonen M. Hirvensalo P. Kulju S. Wenzel C. (2021). Comparative hepatic and intestinal efflux transport of statins. Drug Metab. Dispos. 49 (9), 750–759. 10.1124/dmd.121.000430 34162690

[B14] Fanta S. Jönsson S. Karlsson M. O. Niemi M. Holmberg C. Hoppu K. (2010). Long-term changes in cyclosporine pharmacokinetics after renal transplantation in children: Evidence for saturable presystemic metabolism and effect of NR1I2 polymorphism. J. Clin. Pharmacol. 50 (5), 581–597. 10.1177/0091270009348223 20107201

[B15] Gao Y. Zhang L. Fu Q. (2008). CYP3A4*1G polymorphism is associated with lipid-lowering efficacy of atorvastatin but not of simvastatin. Eur. J. Clin. Pharmacol. 64 (9), 877–882. 10.1007/s00228-008-0502-x 18528690

[B16] Hirota T. Fujita Y. Ieiri I. (2020). An updated review of pharmacokinetic drug interactions and pharmacogenetics of statins. Expert Opin. Drug Metab. Toxicol. 16 (9), 809–822. 10.1080/17425255.2020.1801634 32729746

[B17] Hoenig M. R. Walker P. J. Gurnsey C. Beadle K. Johnson L. (2011). The C3435T polymorphism in ABCB1 influences atorvastatin efficacy and muscle symptoms in a high-risk vascular cohort. J. Clin. Lipidol. 5 (2), 91–96. 10.1016/j.jacl.2011.01.001 21392722

[B18] Hoffart E. Ghebreghiorghis L. Nussler A. K. Thasler W. E. Weiss T. S. Schwab M. (2012). Effects of atorvastatin metabolites on induction of drug-metabolizing enzymes and membrane transporters through human pregnane X receptor. Br. J. Pharmacol. 165, (5), 1595–1608. 10.1111/j.1476-5381.2011.01665.x 21913896 PMC3372740

[B19] Imai Y. Nakane M. Kage K. Tsukahara S. Ishikawa E. Tsuruo T. (2002). C421A polymorphism in the human breast cancer resistance protein gene is associated with low expression of Q141K protein and low-level drug resistance. Mol. Cancer Ther. 1 (8), 611–616. 12479221

[B20] Jemal M. Xia Y. Q. (2000). Bioanalytical method validation design for the simultaneous quantitation of analytes that may undergo interconversion during analysis. J. Pharm. Biomed. Anal. 22 (5), 813–827. 10.1016/s0731-7085(00)00245-4 10815724

[B21] Jungers P. Massy Z. A. Nguyen Khoa T. Fumeron C. Labrunie M. Lacour B. (1997). Incidence and risk factors of atherosclerotic cardiovascular accidents in predialysis chronic renal failure patients: A prospective study. Nephrol. Dial. Transpl. 12 (12), 2597–2602. 10.1093/ndt/12.12.2597 9430858

[B22] Kadam P. Ashavaid T. F. Ponde C. K. Rajani R. M. (2016). Genetic determinants of lipid-lowering response to atorvastatin therapy in an Indian population. J. Clin. Pharm. Ther. 41 (3), 329–333. 10.1111/jcpt.12369 26932749

[B23] Kim K. A. Park P. W. Lee O. J. Kang D. K. Park J. Y. (2007). Effect of polymorphic CYP3A5 genotype on the single-dose simvastatin pharmacokinetics in healthy subjects. J. Clin. Pharmacol. 47 (1), 87–93. 10.1177/0091270006295063 17192506

[B24] Kimchi-Sarfaty C. Oh J. M. Kim I. W. Sauna Z. E. Calcagno A. M. Ambudkar S. V. (2007). A "silent" polymorphism in the MDR1 gene changes substrate specificity. Science 315 (5811), 525–528. 10.1126/science.1135308 17185560

[B25] Knauer M. J. Urquhart B. L. Meyer zu Schwabedissen H. E. Schwarz U. I. Lemke C. J. Leake B. F. (2010). Human skeletal muscle drug transporters determine local exposure and toxicity of statins. Circ. Res. 106 (2), 297–306. 10.1161/circresaha.109.203596 19940267

[B26] Kondo C. Suzuki H. Itoda M. Ozawa S. Sawada J. Kobayashi D. (2004). Functional analysis of SNPs variants of BCRP/ABCG2. Pharm. Res. 21 (10), 1895–1903. 10.1023/b:pham.0000045245.21637.d4 15553238

[B27] König J. Cui Y. Nies A. T. Keppler D. (2000). A novel human organic anion transporting polypeptide localized to the basolateral hepatocyte membrane. Am. J. Physiology-Gastrointestinal Liver Physiology 278 (1), G156–G164. 10.1152/ajpgi.2000.278.1.G156 10644574

[B28] Kopin L. Lowenstein C. (2017). Dyslipidemia. Ann. Intern Med. 167 (11), Itc81–itc96. 10.7326/aitc201712050 29204622

[B29] Kuehl P. Zhang J. Lin Y. Lamba J. Assem M. Schuetz J. (2001). Sequence diversity in CYP3A promoters and characterization of the genetic basis of polymorphic CYP3A5 expression. Nat. Genet. 27 (4), 383–391. 10.1038/86882 11279519

[B30] Lamba J. Lamba V. Schuetz E. (2005). Genetic variants of PXR (NR1I2) and CAR (NR1I3) and their implications in drug metabolism and pharmacogenetics. Curr. Drug Metab. 6 (4), 369–383. 10.2174/1389200054633880 16101575

[B31] Lea A. P. McTavish D. (1997). Atorvastatin. A review of its pharmacology and therapeutic potential in the management of hyperlipidaemias. Drugs 53 (5), 828–847. 10.2165/00003495-199753050-00011 9129869

[B32] Lee N. Maeda K. Fukizawa S. Ieiri I. Tomaru A. Akao H. (2019). Microdosing clinical study to clarify pharmacokinetic and pharmacogenetic characteristics of atorvastatin in Japanese hypercholesterolemic patients. Drug Metab. Pharmacokinet. 34 (6), 387–395. 10.1016/j.dmpk.2019.08.004 31594719

[B33] Lennernäs H. (2003). Clinical pharmacokinetics of atorvastatin. Clin. Pharmacokinet. 42 (13), 1141–1160. 10.2165/00003088-200342130-00005 14531725

[B34] Levey A. S. Stevens L. A. Schmid C. H. Zhang Y. L. Castro A. F. 3rd Feldman H. I. (2009). A new equation to estimate glomerular filtration rate. Ann. Intern Med. 150 (9), 604–612. 10.7326/0003-4819-150-9-200905050-00006 19414839 PMC2763564

[B35] Link E. Parish S. Armitage J. Bowman L. Heath S. Matsuda F. (2008). SLCO1B1 variants and statin-induced myopathy-a genomewide study. N. Engl. J. Med. 359 (8), 789–799. 10.1056/NEJMoa0801936 18650507

[B36] Liu J. Chen Z. Chen H. Hou Y. Lu W. He J. (2017). Genetic polymorphisms contribute to the individual variations of imatinib mesylate plasma levels and adverse reactions in Chinese GIST patients. Int. J. Mol. Sci. 18 (3), 603. 10.3390/ijms18030603 28335376 PMC5372619

[B37] Liyanage T. Toyama T. Hockham C. Ninomiya T. Perkovic V. Woodward M. (2022). Prevalence of chronic kidney disease in Asia: A systematic review and analysis. BMJ Glob. Health 7 (1), e007525. 10.1136/bmjgh-2021-007525 PMC8796212 35078812

[B38] Low S. K. Kiyotani K. Mushiroda T. Daigo Y. Nakamura Y. Zembutsu H. (2009). Association study of genetic polymorphism in ABCC4 with cyclophosphamide-induced adverse drug reactions in breast cancer patients. J. Hum. Genet. 54 (10), 564–571. 10.1038/jhg.2009.79 19696793

[B39] Maeda K. Ikeda Y. Fujita T. Yoshida K. Azuma Y. Haruyama Y. (2011). Identification of the rate-determining process in the hepatic clearance of atorvastatin in a clinical cassette microdosing study. Clin. Pharmacol. Ther. 90 (4), 575–581. 10.1038/clpt.2011.142 21832990

[B40] Mangravite L. M. Thorn C. F. Krauss R. M. (2006). Clinical implications of pharmacogenomics of statin treatment. Pharmacogenomics J. 6 (6), 360–374. 10.1038/sj.tpj.6500384 16550210

[B41] Marino M. di Masi A. Trezza V. Pallottini V. Polticelli F. Ascenzi P. (2011). Xenosensors CAR and PXR at work: Impact on statin metabolism. Curr. Drug Metab. 12 (3), 300–311. 10.2174/138920011795101859 21395534

[B42] Mason R. P. (2006). Molecular basis of differences among statins and a comparison with antioxidant vitamins. Am. J. Cardiol. 98, 34p–41p. 10.1016/j.amjcard.2006.09.018 17126678

[B43] Mbatchi L. C. Gassiot M. Pourquier P. Goberna A. Mahammedi H. Mourey L. (2017). Association of NR1I2, CYP3A5 and ABCB1 genetic polymorphisms with variability of temsirolimus pharmacokinetics and toxicity in patients with metastatic bladder cancer. Cancer Chemother. Pharmacol. 80 (3), 653–659. 10.1007/s00280-017-3379-5 28676933

[B44] Miura M. Satoh S. Inoue K. Kagaya H. Saito M. Inoue T. (2008). Influence of CYP3A5, ABCB1 and NR1I2 polymorphisms on prednisolone pharmacokinetics in renal transplant recipients. Steroids 73 (11), 1052–1059. 10.1016/j.steroids.2008.04.002 18502461

[B45] Palikhe S. Uuganbayar U. Trinh H. K. T. Ban G. Y. Yang E. M. Park H. S. (2017). A role of the ABCC4 gene polymorphism in airway inflammation of asthmatics. Mediat. Inflamm. 2017, 3549375. 10.1155/2017/3549375 PMC5474232 28659663

[B46] Park J. E. Kim K. B. Bae S. K. Moon B. S. Liu K. H. Shin J. G. (2008). Contribution of cytochrome P450 3A4 and 3A5 to the metabolism of atorvastatin. Xenobiotica 38 (9), 1240–1251. 10.1080/00498250802334391 18720283

[B47] Peng C. Ding Y. Yi X. Shen Y. Dong Z. Cao L. (2018). Polymorphisms in CYP450 genes and the therapeutic effect of atorvastatin on ischemic stroke: A retrospective cohort study in Chinese population. Clin. Ther. 40 (3), 469–477.e2. 10.1016/j.clinthera.2018.02.002 29500141

[B48] Prado Y. Zambrano T. Salazar L. A. (2018). Transporter genes ABCG2 rs2231142 and ABCB1 rs1128503 polymorphisms and atorvastatin response in Chilean subjects. J. Clin. Pharm. Ther. 43 (1), 87–91. 10.1111/jcpt.12607 28833323

[B49] Prake-Davis (2004). Prake-davis, Product information: Lipitor (atorvastatin calcium). Ann Arbor, MI: Prake-Davis(Divisioin of Pfizer Inc.

[B50] Prueksaritanont T. Subramanian R. Fang X. Ma B. Qiu Y. Lin J. H. (2002). Glucuronidation of statins in animals and humans: A novel mechanism of statin lactonization. Drug Metab. Dispos. 30 (5), 505–512. 10.1124/dmd.30.5.505 11950779

[B51] Rebecchi I. M. Rodrigues A. C. Arazi S. S. Genvigir F. D. Willrich M. A. Hirata M. H. (2009). ABCB1 and ABCC1 expression in peripheral mononuclear cells is influenced by gene polymorphisms and atorvastatin treatment. Biochem. Pharmacol. 77 (1), 66–75. 10.1016/j.bcp.2008.09.019 18851956

[B52] Riedmaier S. Klein K. Winter S. Hofmann U. Schwab M. Zanger U. M. (2011). Paraoxonase (PON1 and PON3) polymorphisms: Impact on liver expression and atorvastatin-lactone hydrolysis. Front. Pharmacol. 2, 41. 10.3389/fphar.2011.00041 21852972 PMC3147178

[B53] Rogers R. S. Parker A. Vainer P. D. Elliott E. Sudbeck D. Parimi K. (2021). The interface between cell signaling pathways and pregnane X receptor. Cells 10 (11), 3262. 10.3390/cells10113262 34831484 PMC8617909

[B54] Rungtivasuwan K. Avihingsanon A. Thammajaruk N. Mitruk S. Burger D. M. Ruxrungtham K. (2015). Influence of ABCC2 and ABCC4 polymorphisms on tenofovir plasma concentrations in Thai HIV-infected patients. Antimicrob. Agents Chemother. 59 (6), 3240–3245. 10.1128/aac.04930-14 25801567 PMC4432150

[B55] Russel F. G. Koenderink J. B. Masereeuw R. (2008). Multidrug resistance protein 4 (MRP4/ABCC4): A versatile efflux transporter for drugs and signalling molecules. Trends Pharmacol. Sci. 29 (4), 200–207. 10.1016/j.tips.2008.01.006 18353444

[B56] Sánchez-Martín A. Cabrera Figueroa S. Cruz R. Porras-Hurtado L. Calvo-Boyero F. Rasool M. (2016). Gene-gene interactions between DRD3, MRP4 and CYP2B6 polymorphisms and its influence on the pharmacokinetic parameters of efavirenz in HIV infected patients. Drug Metab. Pharmacokinet. 31 (5), 349–355. 10.1016/j.dmpk.2016.06.001 27665700

[B57] Sarnak M. J. Levey A. S. Schoolwerth A. C. Coresh J. Culleton B. Hamm L. L. (2003). Kidney disease as a risk factor for development of cardiovascular disease: A statement from the American heart association councils on kidney in cardiovascular disease, high blood pressure research, clinical cardiology, and Epidemiology and prevention. Circulation 108 (17), 2154–2169. 10.1161/01.Cir.0000095676.90936.80 14581387

[B58] Schirmer M. Rosenberger A. Klein K. Kulle B. Toliat M. R. Nürnberg P. (2007). Sex-dependent genetic markers of CYP3A4 expression and activity in human liver microsomes. Pharmacogenomics 8 (5), 443–453. 10.2217/14622416.8.5.443 17465708

[B59] Swart M. Whitehorn H. Ren Y. Smith P. Ramesar R. S. Dandara C. (2012). PXR and CAR single nucleotide polymorphisms influence plasma efavirenz levels in South African HIV/AIDS patients. BMC Med. Genet. 13, 112. 10.1186/1471-2350-13-112 23173844 PMC3523080

[B60] Tanaka Y. Manabe A. Fukushima H. Suzuki R. Nakadate H. Kondoh K. (2015). Multidrug resistance protein 4 (MRP4) polymorphisms impact the 6-mercaptopurine dose tolerance during maintenance therapy in Japanese childhood acute lymphoblastic leukemia. Pharmacogenomics J. 15 (4), 380–384. 10.1038/tpj.2014.74 25403995

[B61] Teft W. A. Welch S. Lenehan J. Parfitt J. Choi Y. H. Winquist E. (2015). OATP1B1 and tumour OATP1B3 modulate exposure, toxicity, and survival after irinotecan-based chemotherapy. Br. J. Cancer 112 (5), 857–865. 10.1038/bjc.2015.5 25611302 PMC4453959

[B62] Thompson J. F. Man M. Johnson K. J. Wood L. S. Lira M. E. Lloyd D. B. (2005). An association study of 43 SNPs in 16 candidate genes with atorvastatin response. Pharmacogenomics J. 5 (6), 352–358. 10.1038/sj.tpj.6500328 16103896

[B63] Tonelli M. Wanner C. Kidney Disease: Improving Global Outcomes Lipid Guideline Development Work Group Members (2014). Lipid management in chronic kidney disease: Synopsis of the kidney disease: Improving global Outcomes 2013 clinical practice guideline. Ann. Intern Med. 160 (3), 182. 10.7326/m13-2453 24323134

[B64] Turner R. M. Fontana V. FitzGerald R. Morris A. P. Pirmohamed M. (2020a). Investigating the clinical factors and comedications associated with circulating levels of atorvastatin and its major metabolites in secondary prevention. Br. J. Clin. Pharmacol. 86 (1), 62–74. 10.1111/bcp.14133 31656041 PMC6983514

[B65] Turner R. M. Fontana V. Zhang J. E. Carr D. Yin P. FitzGerald R. (2020b). A genome-wide association study of circulating levels of atorvastatin and its major metabolites. Clin. Pharmacol. Ther. 108 (2), 287–297. 10.1002/cpt.1820 32128760

[B66] Venkatasubramanian R. Fukuda T. Niu J. Mizuno T. Chidambaran V. Vinks A. A. (2014). ABCC3 and OCT1 genotypes influence pharmacokinetics of morphine in children. Pharmacogenomics 15 (10), 1297–1309. 10.2217/pgs.14.99 25155932 PMC4190075

[B67] Yeung C. K. Shen D. D. Thummel K. E. Himmelfarb J. (2014). Effects of chronic kidney disease and uremia on hepatic drug metabolism and transport. Kidney Int. 85 (3), 522–528. 10.1038/ki.2013.399 24132209 PMC4276411

[B68] Zeng G. Wang L. Shi L. Li H. Zhu M. Luo J. (2020). Variability of voriconazole concentrations in patients with hematopoietic stem cell transplantation and hematological malignancies: Influence of loading dose, procalcitonin, and pregnane X receptor polymorphisms. Eur. J. Clin. Pharmacol. 76 (4), 515–523. 10.1007/s00228-020-02831-1 31932875

[B69] Zhang J. Kuehl P. Green E. D. Touchman J. W. Watkins P. B. Daly A. (2001). The human pregnane X receptor: Genomic structure and identification and functional characterization of natural allelic variants. Pharmacogenetics 11 (7), 555–572. 10.1097/00008571-200110000-00003 11668216

[B70] Zhou Y. Ingelman-Sundberg M. Lauschke V. M. (2017). Worldwide distribution of cytochrome P450 alleles: A meta-analysis of population-scale sequencing projects. Clin. Pharmacol. Ther. 102 (4), 688–700. 10.1002/cpt.690 28378927 PMC5600063

[B71] Zubiaur P. Benedicto M. D. Villapalos-García G. Navares-Gómez M. Mejía-Abril G. Román M. (2021). SLCO1B1 phenotype and CYP3A5 polymorphism significantly affect atorvastatin bioavailability. J. Personalized Med. 11 (3), 204. 10.3390/jpm11030204 PMC7999651 33805706

