# Supplementary material for: Effect of polymorphisms in drug metabolism and transportation on plasma concentration of atorvastatin and its metabolites in patients with chronic kidney disease
Source: Front Pharmacol. 2023 Feb 27;14:1102810. doi: 10.3389/fphar.2023.1102810 (PMC10010391; doi:10.3389/fphar.2023.1102810)
Supplement: Supplementary file 1 [file DataSheet1.docx]

**Supplementary Table S1** The inclusion and exclusion criteria

| Inclusion criteria | 1. Aged≥ 50 years, eGFR < 60 mL·min-^1^·1.73m^-2^ non-dialysis and kidney transplant patients;  2. Aged≥50, eGFR ≥ 60 mL·min^-1^·1.73m^-2^ chronic kidney disease patients;  3. CKD patients aged 18to49 years, without dialysis or kidney transplants, having with one of the following:  a. coronary artery disease (myocardial infarction or coronary remodeling); b. diabetes; c. the estimated incidence of coronary death or nonfatal myocardial infarction within 10 years is more than 10%; d. ischemic stroke.  4. Dialysis for patients with CKD, LDL-C > 3.37mmol/L.  In accordance with any of the above, it would be included in the study. |
| --- | --- |
| Exclusion criteria | 1. Pregnant and breastfeeding women;  2. Patients with active liver disease;  3. Allergic to statins;  4. The patients are already taking statins;  5. Taking other lipid-lowering drugs: nicotinic acid, fibrates and their derivatives, probucol, fish oil, bile acid binding resin;  6. Drugs that may increase the risk of myopathy: cyclosporine, human immunodeficiency virus (HIV) protease inhibitors (tipranavir + ritonavir, lopinavir + ritonavir, saquinavir + ritonavir, darunavir + ritonavir, foxanavir, foxanavir + ritonavir, nelfinavir), hepatitis C protease inhibitor (telaprevir and boceprevir), clarithromycin, itraconazole;  7. Other co-administered drugs that may affect blood biochemical measurements: HIV protease inhibitors (e.g. lopinavir, darunavir, ritonavir), azole antifungals (e.g. itraconazole, ketoconazole), macrolide anti-infectives (e.g. erythromycin, clarithromycin, telithromycin), fibrates (e.g. gemfibert, benzofibrate), nicotinic acid, nefazodone, cyclosporine, amiodarone, diltiazem, fiducic acid, etc.  8. Diseases of biliary depression;  9. Diseases of the haematological system;  10. Neoplastic Diseases;  11. Diseases of the gastrointestinal tract;  12. Hypothyroidism  Any of the above conditions can be ruled out. |

**Supplementary Table S2** The allelic frequencies of SNPs and HWE test.(n=210)

| Gene | SNPs | Allelic frequencies | | | CHS (MAF) | P_1_-value | P_2_-value |
| --- | --- | --- | --- | --- | --- | --- | --- |
| *CYP3A4* | rs2242480 | C  0.688 |  | T  0.312 | T (0.238) | 0.855 | 0.962 |
|  | rs4646437 | A  0.193 |  | G  0.807 | A (0.124) | 0.063 | 0.114 |
| *CYP3A5* | rs776746 | C  0.526 |  | T  0.474 | T (0.271) | 0.003 | 0.000 |
| *SLCO1B1* | rs4149056 | C  0.114 |  | T  0.886 | C (0.119) | 0.391 | 0.441 |
|  | rs2306283 | A  0.202 |  | G  0.798 | A (0.205) | 0.55 | 0.326 |
| *ABCB1* | rs1128503 | A  0.69 |  | G  0.31 | G (0.367) | 0.494 | 0.241 |
|  | rs1045642 | A  0.412 |  | G  0.588 | A (0.305) | 0.187 | 0.214 |
|  | rs2032582 | C  0.486 | A  0.414 | T  0.1 | T (0.129) | 0.027 | 0.000 |
| *ABCG2* | rs2231142 | G  0.631 |  | T  0.369 | T (0.257) | 0.097 | 0.06 |
| *ABCC3* | rs4793665 | C  0.131 |  | T  0.869 | C (0.119) | 0.396 | 0.628 |
|  | rs3742106 | A  0.486 |  | C  0.514 | A (0.486) | 0.071 | 0.131 |
| *ABCC4* | rs9561778 | G  0.669 |  | T  0.331 | T (0.29) | 0.533 | 0.295 |
|  | rs2274407 | A  0.152 |  | C  0.848 | A (0.162) | 0.947 | 0.948 |
|  | rs868853 | C  0.129 |  | T  0.871 | C (0.133) | 0.346 | 0.968 |
| *ABCC5* | rs562 | C  0.493 |  | T  0.507 | T (0.457) | 0.167 | 0.182 |
|  | rs3749438 | A  0.436 |  | G  0.564 | A (0.39) | 0.97 | 0.453 |
| *NR1l2* | rs6785049 | A  0.412 |  | G  0.588 | A (0.381) | 0.126 | 0.716 |
|  | rs1523127 | A  0.771 |  | C  0.229 | C (0.229) | 0.12 | 0.966 |

Abbreviations: HWE, Hardy–Weinberg equilibrium; SNP, single nucleotide polymorphism; CHS, Chinese Han Southern; MAF, minor allele frequency.P_1_: HWE test, P_2_: compare to CHS using the Chi-square test.

**Supplementary Table S3** Effect of baseline clinical characteristics on plasma concentration of atorvastatin and its metabolites (A)

| characteristics | mean±SD/  N (%) | AT+ATL, μmol/L | | | | 2-AT+2-ATL, μmol/L | | | |
| --- | --- | --- | --- | --- | --- | --- | --- | --- | --- |
|  |  | Univariate Analysis | | Multivariate Analysis | | Univariate Analysis | | Multivariate Analysis | |
|  |  | Estimate | P-value | Estimate | P-value | Estimate | P-value | Estimate | P-value |
| gender，male | 116 (54.7) | -0.104 | 0.262 |  |  | -0.07 | 0.244 |  |  |
| age，years | 56.4±10.9 | 0.003 | 0.507 |  |  | -0.003 | 0.264 |  |  |
| BMI，kg/m^2^ | 23.2±4.64 | -0.003 | 0.854 |  |  | -0.001 | 0.891 |  |  |
| SP，mmHg | 142.0±30.0 | 0.004 | 0.119 |  |  | 0.002 | 0.183 |  |  |
| DP，mmHg | 85.5±12.0 | 0.005 | 0.173 |  |  | 0.002 | 0.466 |  |  |
| TC，mmol/L | 5.89±1.86 | 0.001 | 0.96 |  |  | -0.009 | 0.586 |  |  |
| TG，mmol/L | 2.08±1.72 | -0.021 | 0.425 |  |  | -0.015 | 0.377 |  |  |
| HDL，mmol/L | 1.26±0.41 | 0.275 | **0.022** |  |  | 0.139 | 0.078 |  |  |
| LDL，mmol/L | 3.86±1.29 | -0.037 | 0.338 |  |  | -0.03 | 0.219 |  |  |
| ApoAI，g/L | 1.40±0.33 | 0.421 | **0.003** | 0.421 | **0.003** | 0.173 | 0.064 |  |  |
| ApoB100，g/L | 1.09±0.34 | -0.114 | 0.414 |  |  | -0.081 | 0.357 |  |  |
| LPa，mg/L | 292.75±255.75 | 2.57E-04 | 0.161 |  |  | 2.45E-04 | **0.039** | 2.44E-04 | **0.037** |
| ALT，U/L | 18.70±12.58 | -0.002 | 0.68 |  |  | 1.53E-04 | 0.949 |  |  |
| AST，U/L | 20.50±8.39 | 0.001 | 0.867 |  |  | 0.002 | 0.587 |  |  |
| ALP，U/L | 83.23±31.56 | 0.001 | 0.333 |  |  | 0.001 | 0.341 |  |  |
| CHE，KU/L | 7.44±2.08 | -0.017 | 0.441 |  |  | -0.001 | 0.947 |  |  |
| GGT，U/L | 34.59±30.62 | 0.002 | 0.103 |  |  | 0.001 | 0.476 |  |  |
| TBIL，μmol/L | 8.80±3.20 | 5.58E-05 | 0.997 |  |  | 0.004 | 0.712 |  |  |
| IBIL，μmol/L | 7.40±2.67 | -0.004 | 0.803 |  |  | 0.003 | 0.808 |  |  |
| ALB，g/L | 36.58±6.86 | -0.01 | 0.142 |  |  | -0.008 | 0.076 |  |  |
| GLB，g/L | 32.78±5.62 | 4.63E-05 | 0.996 |  |  | -0.003 | 0.523 |  |  |
| Glu，mmol/L | 6.66±3.60 | -0.023 | 0.079 |  |  | -0.019 | **0.028** | -0.019 | **0.027** |
| Cr，mmol/L | 346.20±212.65 | 3.13E-04 | 0.148 |  |  | 0.00016 | 0.251 |  |  |
| Urea，mmol/L | 16.08±9.05 | 0.006 | 0.258 |  |  | 0.003 | 0.424 |  |  |
| UA，μmol/L | 500.37±126.01 | -0.0001 | 0.784 |  |  | 4.40E-05 | 0.85 |  |  |
| CKMB，U/L | 14.00±6.92 | 0.007 | 0.328 |  |  | 0.002 | 0.605 |  |  |
| HBDH，U/L | 145.04±43.76 | 0.002 | 0.093 |  |  | 0.001 | 0.205 |  |  |
| LDH，U/L | 217.06±68.13 | 0.001 | 0.224 |  |  | 3.42E-04 | 0.434 |  |  |
| CK，U/L | 140.32±163.77 | -1.57E-06 | 0.996 |  |  | -8.46E-05 | 0.634 |  |  |
| hisCRP，mg/L | 8.00±18.00 | 0.003 | 0.203 |  |  | 0.003 | 0.082 |  |  |
| eGFR，ml/min/1.73m^2^ | 24.16±19.14 | -0.002 | 0.312 |  |  | -4.76E-04 | 0.76 |  |  |
| Smoking | 61 (28.9) | -0.029 | 0.778 |  |  | -0.029 | 0.654 |  |  |
| Alcohol consumption | 14 (6.60 | -0.301 | 0.099 |  |  | -0.175 | 0.146 |  |  |
| Hypertension | 167 (79.5) | -0.02 | 0.864 |  |  | -0.055 | 0.466 |  |  |
| Diabetes | 87 (42.) | -0.041 | 0.665 |  |  | -0.047 | 0.439 |  |  |
| Cardiovascular diseases | 53 (29.1) | -0.019 | 0.856 |  |  | 0.026 | 0.71 |  |  |

**Supplementary Table S3** Effect of baseline clinical characteristics on plasma concentration of atorvastatin and its metabolites (B)

| characteristics | mean±SD/  N(%) | 4-AT+4-ATL, μmol/L | | | | AT+2-AT+4-AT, μmol/L | | | |
| --- | --- | --- | --- | --- | --- | --- | --- | --- | --- |
|  |  | Univariate Analysis | | Multivariate Analysis | | Univariate Analysis | | Multivariate Analysis | |
|  |  | Estimate | P-value | Estimate | P-value | Estimate | P-value | Estimate | P-value |
| gender，male | 116（54.7） | -0.036 | 0.608 |  |  | -0.084 | 0.205 |  |  |
| age，years | 56.4±10.9 | -0.01 | 0.671 |  |  | -0.003 | 0.258 |  |  |
| BMI，kg/m^2^ | 23.2±4.64 | 0.008 | 0.471 |  |  | -0.002 | 0.872 |  |  |
| SP，mmHg | 142.0±30.0 | 0.001 | 0.586 |  |  | 0.002 | 0.185 |  |  |
| DP，mmHg | 85.5±12.0 | 0.001 | 0.776 |  |  | 0.001 | 0.613 |  |  |
| TC，mmol/L | 5.89±1.86 | -0.013 | 0.522 |  |  | 0.007 | 0.699 |  |  |
| TG，mmol/L | 2.08±1.72 | -0.034 | 0.089 |  |  | -0.028 | 0.148 |  |  |
| HDL，mmol/L | 1.26±0.41 | 0.094 | 0.309 |  |  | 0.175 | **0.043** |  |  |
| LDL，mmol/L | 3.86±1.29 | -0.016 | 0.565 |  |  | 0.004 | 0.89 |  |  |
| ApoAI，g/L | 1.40±0.33 | 0.213 | 0.054 | 0.264 | **0.014** | 0.278 | **0.007** | 0.374 | **<0.001** |
| ApoB100，g/L | 1.09±0.34 | -0.045 | 0.664 |  |  | 0.022 | 0.829 |  |  |
| LPa，mg/L | 292.75±255.75 | 4.17E-04 | **0.002** | 3.08E-04 | **0.02** | 3.17E-04 | **0.016** |  |  |
| ALT，U/L | 18.70±12.58 | -0.001 | 0.838 |  |  | -0.003 | 0.272 |  |  |
| AST，U/L | 20.50±8.39 | 0.002 | 0.636 |  |  | -0.003 | 0.524 |  |  |
| ALP，U/L | 83.23±31.56 | 2.36E-04 | 0.829 |  |  | 0.001 | 0.495 |  |  |
| CHE，KU/L | 7.44±2.08 | -0.009 | 0.609 |  |  | -0.003 | 0.828 |  |  |
| GGT，U/L | 34.59±30.62 | 2.04E-04 | 0.855 |  |  | -5.97E-05 | 0.956 |  |  |
| TBIL，μmol/L | 8.80±3.20 | 0.008 | 0.482 |  |  | -0.008 | 0.464 |  |  |
| IBIL，μmol/L | 7.40±2.67 | 0.008 | 0.529 |  |  | -0.01 | 0.421 |  |  |
| ALB，g/L | 36.58±6.86 | -0.004 | 0.476 |  |  | -0.01 | 0.056 | -0.013 | **0.012** |
| GLB，g/L | 32.78±5.62 | 0.003 | 0.615 |  |  | 0.001 | 0.805 |  |  |
| Glu，mmol/L | 6.66±3.60 | -0.023 | **0.021** | -0.021 | **0.021** | -0.024 | **0.013** | -0.023 | **0.012** |
| Cr，mmol/L | 346.20±212.65 | 2.01E-04 | 0.218 |  |  | 2.99E-04 | 0.055 |  |  |
| Urea，mmol/L | 16.08±9.05 | 0.001 | 0.727 |  |  | 0.006 | 0.115 |  |  |
| UA，μmol/L | 500.37±126.01 | 1.76E-05 | 0.949 |  |  | 2.47E-04 | 0.346 |  |  |
| CKMB，U/L | 14.00±6.92 | 0.003 | 0.518 |  |  | 0.005 | 0.286 |  |  |
| HBDH，U/L | 145.04±43.76 | 0.001 | 0.374 |  |  | 0.002 | **0.031** |  |  |
| LDH，U/L | 217.06±68.13 | 4.04E-04 | 0.433 |  |  | 0.001 | 0.085 |  |  |
| CK，U/L | 140.32±163.77 | -1.31E-04 | 0.529 |  |  | -1.42E-04 | 0.478 |  |  |
| hisCRP，mg/L | 8.00±18.00 | 0.006 | **0.008** | 0.006 | **0.004** | 0.003 | 0.074 | 0.003 | 0.099 |
| eGFR，ml/min/1.73m^2^ | 24.16±19.14 | -0.002 | 0.225 |  |  | -0.003 | **0.044** | -0.004 | **0.031** |
| Smoking | 61（28.9） | -0.063 | 0.418 |  |  | 0.016 | 0.834 |  |  |
| Alcohol consumption | 14（6.6） | -0.391 | **0.005** | -0.375 | **0.004** | -0.273 | **0.038** | -0.303 | **0.015** |
| Hypertension | 167（79.5） | 0.024 | 0.783 |  |  | 0.031 | 0.715 |  |  |
| Diabetes | 87（42.6） | -0.106 | 0.134 |  |  | -0.086 | 0.205 |  |  |
| Cardiovascular diseases | 53（29.1） | 0.062 | 0.44 |  |  | 0.088 | 0.248 |  |  |

**Supplementary Table S4A** Association analysis between SNP and plasma concentration of atorvastatin and its metabolites in different genotypes.

| SNP | Gene | P-value | | | |
| --- | --- | --- | --- | --- | --- |
|  |  | AT+ATL | 2-AT+2-ATL | 4-AT+4-ATL | AT+2-AT+4-AT |
| rs1128503 | *ABCB1* | 0.113 | 0.167 | 0.616 | 0.477 |
| rs2231142 | *ABCG2* | 0.269 | 0.572 | 0.736 | 0.979 |
| rs2242480 | *CYP3A4* | 0.91 | 0.509 | 0.1 | 0.741 |
| rs776746 | *CYP3A5* | 0.14 | 0.089 | 0.124 | 0.175 |
| rs2306283 | *SLCO1B1* | 0.336 | 0.755 | 0.686 | 0.881 |
| rs4149056 | *SLCO1B1* | 0.402 | 0.246 | 0.832 | 0.806 |
| rs1045642 | *ABCB1* | 0.862 | 0.866 | 0.991 | 0.913 |
| rs4646437 | *CYP3A4* | 0.912 | 0.461 | 0.099 | 0.869 |
| rs2032582 | *ABCB1* | 0.521 | 0.415 | 0.706 | 0.322 |
| **rs3742106** | *ABCC4* | 0.068 | **0.021** | 0.278 | **0.02** |
| rs562 | *ABCC5* | 0.345 | 0.373 | 0.704 | 0.413 |
| rs3749438 | *ABCC5* | 0.984 | 0.737 | 0.555 | 0.962 |
| **rs868853** | *ABCC4* | 0.387 | **0.012** | 0.088 | 0.061 |
| rs4793665 | *ABCC3* | 0.84 | 0.901 | 0.332 | 0.593 |
| **rs9561778** | *ABCC4* | **0.042** | 0.116 | 0.301 | 0.623 |
| rs1523127 | *NR1l2* | 0.265 | 0.191 | 0.66 | 0.791 |
| rs2274407 | *ABCC4* | 0.847 | 0.884 | 0.688 | 0.668 |
| rs6785049 | *NR1l2* | 0.199 | 0.138 | 0.123 | 0.192 |

**Supplementary Table S4B** Association analysis between SNP and plasma concentration of atorvastatin and its metabolites in dominant model

| SNP | Gene | Dominant，P-value | | | |
| --- | --- | --- | --- | --- | --- |
|  |  | AT+ATL | 2-AT+2-ATL | 4-AT+4-ATL | AT+2-AT+4-AT |
| rs1128503 | *ABCB1* | 0.235 | 0.517 | 0.915 | 0.555 |
| rs2231142 | *ABCG2* | 0.313 | 0.765 | 0.931 | 0.917 |
| rs2242480 | *CYP3A4* | 0.732 | 0.395 | 0.059 | 0.692 |
| rs776746 | *CYP3A5* | 0.113 | 0.084 | 0.07 | 0.09 |
| rs2306283 | *SLCO1B1* | 0.146 | 0.475 | 0.989 | 0.885 |
| rs4149056 | *SLCO1B1* | 0.229 | 0.1 | 0.574 | 0.95 |
| rs1045642 | *ABCB1* | 0.795 | 0.732 | 0.94 | 0.832 |
| rs4646437 | *CYP3A4* | 0.714 | 0.255 | **0.041** | 0.609 |
| rs2032582 | *ABCB1* | 0.643 | 0.601 | 0.769 | 0.519 |
| **rs3742106** | *ABCC4* | **0.034** | **0.015** | 0.378 | 0.11 |
| rs562 | *ABCC5* | 0.165 | 0.172 | 0.724 | 0.257 |
| rs3749438 | *ABCC5* | 0.949 | 0.508 | 0.773 | 0.91 |
| **rs868853** | *ABCC4* | 0.384 | **0.017** | 0.098 | 0.276 |
| rs4793665 | *ABCC3* | 0.924 | 0.719 | 0.897 | 0.496 |
| **rs9561778** | *ABCC4* | **0.039** | 0.059 | 0.157 | 0.337 |
| rs1523127 | *NR1l2* | 0.447 | 0.53 | 0.456 | 0.693 |
| rs2274407 | *ABCC4* | 0.649 | 0.682 | 0.394 | 0.377 |
| **rs6785049** | *NR1l2* | 0.161 | **0.048** | 0.074 | 0.093 |

**Supplementary Table S4C** Association analysis between SNP and plasma concentration of atorvastatin and its metabolites in recessive model

| SNP | Gene | Recessive，P-value | | | |
| --- | --- | --- | --- | --- | --- |
|  |  | AT+ATL | 2-AT+2-ATL | 4-AT+4-ATL | AT+2-AT+4-AT |
| **rs1128503** | *ABCB1* | **0.047** | 0.059 | 0.333 | 0.235 |
| rs2231142 | *ABCG2* | 0.343 | 0.372 | 0.468 | 0.843 |
| rs2242480 | *CYP3A4* | 0.879 | 0.308 | 0.119 | 0.45 |
| **rs776746** | *CYP3A5* | 0.079 | **0.049** | 0.101 | 0.152 |
| rs2306283 | *SLCO1B1* | 0.846 | 0.66 | 0.408 | 0.615 |
| rs4149056 | *SLCO1B1* | 0.802 | 0.48 | 0.721 | 0.518 |
| rs1045642 | *ABCB1* | 0.702 | 0.62 | 0.935 | 0.768 |
| rs4646437 | *CYP3A4* | 0.939 | 0.391 | 0.186 | 0.965 |
| rs2032582 | *ABCB1* | 0.404 | 0.328 | 0.404 | 0.282 |
| **rs3742106** | *ABCC4* | 0.133 | **0.046** | 0.126 | **0.008** |
| rs562 | *ABCC5* | 0.98 | 0.47 | 0.534 | 0.745 |
| rs3749438 | *ABCC5* | 0.856 | 0.865 | 0.279 | 0.78 |
| rs868853 | *ABCC4* | 0.441 | 0.322 | 0.373 | 0.09 |
| rs4793665 | *ABCC3* | 0.59 | 0.859 | 0.158 | 0.575 |
| rs9561778 | *ABCC4* | 0.444 | 0.79 | 0.309 | 0.67 |
| rs1523127 | *NR1l2* | 0.216 | 0.128 | 0.504 | 0.646 |
| rs2274407 | *ABCC4* | 0.653 | 0.869 | 0.732 | 0.945 |
| rs6785049 | *NR1l2* | 0.117 | 0.346 | 0.788 | 0.991 |
